# Heterochromatin boundaries maintain centromere position, size and number

**DOI:** 10.1038/s41594-025-01706-2

**Published:** 2025-11-25

**Authors:** Ben L. Carty, Danilo Dubocanin, Marina Murillo-Pineda, Marie Dumont, Emilia Volpe, Pawel Mikulski, Julia Humes, Oliver Whittingham, Daniele Fachinetti, Simona Giunta, Nicolas Altemose, Lars E. T. Jansen

**Affiliations:** 1 Dept of Biochemistry, University of Oxford, Oxford, UK; 2 Department of Genetics, Stanford University, Palo Alto, CA USA; 3 UMR144 and UMR3664, CNRS, Institut Curie, PSL Research University, Sorbonne Université, Paris, France; 4 Laboratory of Genome Evolution, Department of Biology and Biotechnologies ‘Charles Darwin’, University of Rome ‘La Sapienza’, Rome, Italy; 5 Present Address: Infectious Disease and Microbiology Unit. Biomedical Innovation and Research Institute of Cádiz (INiBICA), University Hospital of Jerez, University of Cádiz, Cádiz, Spain; 6 Present Address: Nencki Institute of Experimental Biology, Polish Academy of Sciences, Warsaw, Poland

**Keywords:** DNA methylation, Epigenomics, Chromosome segregation, Histone post-translational modifications, Centromeres

## Abstract

Centromeres are defined by a unique single chromatin domain featuring the histone H3 variant, centromere protein A (CENP-A), and ensure proper chromosome segregation. Centromeric chromatin typically occupies a small subregion of low DNA methylation within multimegabase arrays of hypermethylated alpha-satellite repeats and constitutive pericentric heterochromatin. Here, we define the molecular basis of how heterochromatin serves as a primary driver of centromere and neocentromere position, size and number. Using single-molecule epigenomics, we uncover roles for H3K9me3 methyltransferases SUV39H1/H2 and SETDB1, in addition to noncanonical roles for SUZ12, in maintaining H3K9me3 boundaries at centromeres. Loss of these heterochromatin boundaries leads to the progressive expansion and/or repositioning of the primary CENP-A domain, erosion of surrounding DNA methylation and nucleation of additional functional CENP-A domains across the same alpha-satellite sequences. Our study identifies the functional importance and specialization of different H3K9 methyltransferases across centromeric and pericentric domains, crucial for maintaining centromere domain size and suppressing ectopic centromere nucleation events.

## Abstract (web summary)

Carty et al. identify the H3K9 methyltransferases that restrict the size and position of the centromere protein A chromatin domain, maintaining functional centromeres.

## Main

Centromeres are the chromosomal loci that drive chromosome segregation during mitosis and meiosis^1,2^. Human centromeres are characterized by multimegabase arrays of A+T-rich alpha-satellite DNA^3,4^. Intriguingly, these sequences, while abundant, are not strictly required for centromere function. Instead, centromere position in humans is, to a large extent, defined epigenetically. The key centromere-defining element is the histone H3 variant centromere protein A (CENP-A), which is sufficient to induce de novo centromere formation and appears as a central node in a self-propagating feedback loop that maintains centromere position^5–7^.

The discovery of spontaneous neocentromeres in humans epitomizes the epigenetic nature of centromeres, nucleating at sites devoid of alphoid DNA^8–12^. Once formed, these neocentromeres are functional and propagated indefinitely in somatic cells and, in certain cases, are even transgenerationally inherited^8,12,13^. Moreover, by deleting the endogenous centromere locus in model systems, neocentromeres can be induced experimentally, which rescues chromosome stability^14–18^. In human cells, this approach allows us to explore the genetic and epigenetic contributions to centromere seeding and inheritance, as well as monitor the evolution of spontaneously formed centromeres.

Centromeres are propagated by the stable transmission of CENP-A chromatin that serves as a template for the self-directed assembly of new CENP-A nucleosomes each cell cycle^19–21^. This self-propagating cycle is predicted to generate variation in the precise positioning of the centromere because of the dynamic nature of histone recycling during DNA replication and transcription^22^. Indeed, centromere drift has been observed in evolutionary new centromeres of donkeys and chicken Z chromosomes^23,24^. However, despite this local drift, vertebrate centromeres remain restricted to their broader chromosomal locus even if CENP-A is temporarily removed^25^ and their size remains restricted to ~100–500 kb at both canonical and neocentromeres^3,18,26,27^. The tightly constrained CENP-A domain is somewhat surprising as such ‘islands’ of CENP-A typically reside in megabase-sized domains of homogenous alphoid repeats, indicating the existence of non-sequence-defined centromere boundaries.

One potential boundary is 5mCG DNA methylation. Endogenous human centromeres occur within arrays of alpha-satellite higher-order repeat (HORs) that generally have hypermethylated DNA, except for a distinct site of hypomethylation coinciding with strong enrichment of CENP-A chromatin, termed centromere dip regions (CDRs)^3,28^. In addition, constitutive heterochromatin, marked by histone H3 lysine 9 trimethylation (H3K9me3), spans human satellite (HSat) DNA families in flanking pericentromeric regions, such as the classical HSats 1–3 (refs. ^29,30^), and has been hypothesized to maintain centromere position^31^.

H3K9me3 has also been observed to at least partially overlap with centromeric repeats^32–36^ and was recently mapped within active alpha-satellite HOR arrays^37^, indicating that the chromatin environment at and around the CENP-A locus may be of functional importance. Whether heterochromatin forms a true functional boundary and how this is regulated remain open questions.

## Results

### The CENP-A domain at a nascent human neocentromere drifts while maintaining overall size

**Fig. 1:**
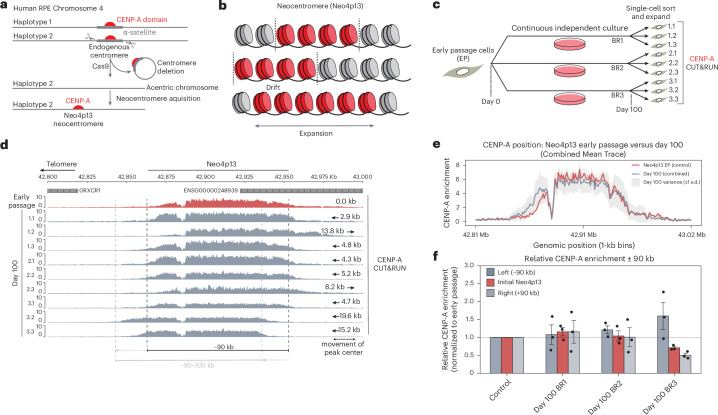
Long-term inheritance and size control of a human neocentromere. **a**, Schematic of the generation of RPE1-Neo4p13, as previously described^18^. **b**, Schematic predictions of centromere inheritance behavior over time. Centromeres may stay stationary, move left or right with tight size and boundary control or gradually expand over time. **c**, Schematic of long-term culture assay to monitor inheritance of Neo4p13, culturing early-passage Neo4p13 for 100 days, followed by single-cell sorting and CENP-A CUT&RUN. BR, biological replicate. **d**, CENP-A CUT&RUN of early-passage Neo4p13 (red) versus 100-day clones (gray). Black dotted lines represent early-passage centromere position. The light-gray dotted line represents the position of clone 3.2 as an example of centromere shift. The degree of movement is indicated for each clone (arrows) **e**, Quantitation of CENP-A enrichment across the genomic region 42.8–43.0 Mb (Chr4 Hap2) represented in 1-kb bins. The red trace represents the control sample (Neo4p13), highlighting early-passage CENP-A enrichment. The gray trace represents the combined mean enrichment for three groups of day 100 clones, BR1, BR2 and BR3, with day 100 ± 1 s.d. (light gray) **f**, Relative CENP-A enrichment in three genomic regions: the initial Neo4p13 locus (defined as a 90-kb window from 42.868 to 42.958 Mb) and immediate flanking regions, 90 kb upstream (‘left (−90 kb)’) and downstream (‘Right (+90 kb)’). Enrichment values are normalized to the early-passage control sample, represented by a baseline of 1 (dashed line). Data are presented as the mean values ± s.e.m. (three BRs). Bar colors highlight the initial Neo4p13 locus (red, opaque) and the outside regions (grays). Source data

We previously isolated a de novo human neocentromere in cultured human RPE1 cells (Fig. 1a)^18^. This neocentromere, on the p arm of Chr4, Neo4p13, is 90 kb in size and formed in a gene-poor region, within high levels of H3K9me3 constitutive heterochromatin^18^, analogous to canonical centromeres. This system offers a unique time-stamped centromere to understand how centromere position and size are maintained, following its formation, independent of satellite sequences (Fig. 1b). We took an early Neo4p13 isolate and proliferated three independent populations for 100 days. We then randomly isolated three clonal subpopulations from each independently evolved pool and assessed centromere position using CENP-A CUT&RUN (Fig. 1c). In early-passage cells (red), CENP-A mapped to a 90-kb window from 42.86 Mb to 42.96 Mb (Fig. 1d, red track), positioned in the same location as the initial neocentromere isolate^18^. Upon prolonged cell culture (day 100, gray), we observed a gradual centromere drift over time (Fig. 1d–f), with shifts in either direction and an average shift of peak center of 8.74 kb (Fig. 1d, arrows). This gradual movement of the CENP-A domain is consistent with previous studies in evolutionary new centromeres^23,24,38^. Strikingly, however, the overall size of the Neo4p13 locus remained relatively tight at approximately 90–100 kb even as this centromere moved (Fig. 1d,e). As the CENP-A domain drifts in one direction, CENP-A nucleosomes are depleted from the trailing end of the domain (for example, Fig. 1d, clones 1.2 and 3.2). These observations suggest that mechanisms exist to tightly maintain centromere boundaries and size of this domain.

### Seeding of Neo4p13 induces local depletion of 5mC and H3K9me3

**Fig. 2:**
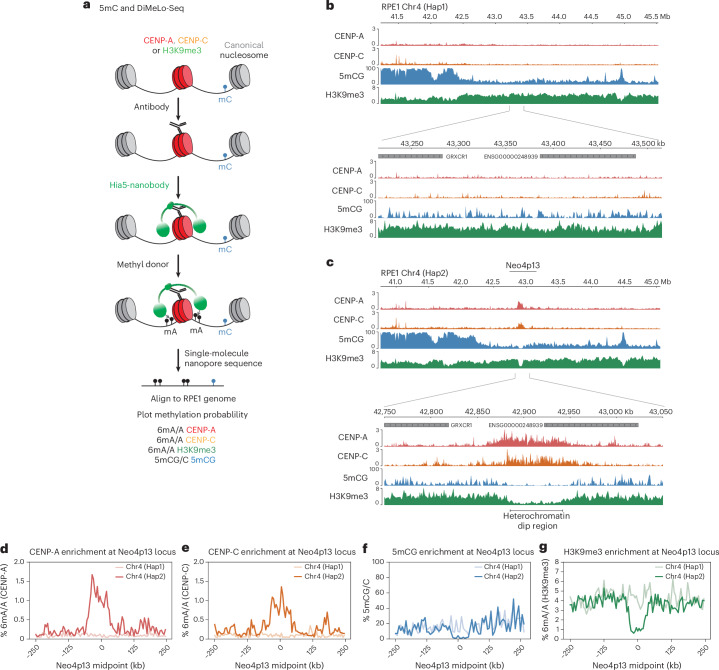
Neo4p13 occupies a distinct H3K9me3-mediated heterochromatin dip on Chr4 Hap2. **a**, Schematic of the workflow of DiMelo-Seq and mapping to the diploid RPE1 genome. **b**,**c**, Enrichment of CENP-A (red), CENP-C (orange), H3K9me3 (green) and 5mCG (blue) at the genomic locus of Neo4p13, mapped to RPE1 Chr4 Hap1 (**b**) and Chr4 Hap2 (**c**). Enrichment of CENP-A/CENP-C and dips in H3K9me3 and 5mCG are specific to Chr4 Hap2. **d**–**g**, CENP-A enrichment (**d**; % 6mA/A, red), CENP-C enrichment (**e**; % 6mA/A, orange), % 5mCG/C (**f**; blue) and H3K9me3 (**g**; % 6mA/A, green) at the Neo4p13 locus for two haplotypes: Chr4 (Hap1) and Chr4 (Hap2). Data are aggregated across a 500-kb region centered at the Neo4p13 coordinates, with flanking regions extending 250 kb on either side. Mean enrichment values are calculated in 100 evenly spaced bins per region. The *x* axis represents the genomic position relative to the midpoint (in kb), while the *y* axis indicates the % 6mA/A or 5mCG/C.

To understand how neocentromere position is maintained, we mapped the local chromatin environment surrounding the CENP-A domain. The RPE1-Neo4p13 cells are diploid, effectively carrying two distinct centromere epialleles, one at the canonical centromeric alpha-satellites of chromosome 4 and one at 4p13 (Fig. 1a). To accurately map chromatin features in a haplotype-specific manner, selectively at the neocentromere allele, we used Oxford Nanopore Technologies (ONT)-based ultralong-read DiMelo-seq^37^, in combination with the cell-line-specific RPE1 reference genome^39^ (Fig. 2a). Neo4p13 maps to Chr4 haplotype 2 (Hap2), indicated by the presence of a 6mA peak at p13 on Hap2 specifically directed toward CENP-A and CENP-C (Fig. 2b–e). Consistent with our previous observations using ChIP-seq^18^, this locus is enriched with H3K9me3 on both haplotypes. Interestingly, seeding of the CENP-A domain resulted in the selective loss of H3K9me3 specifically at the neocentromere haplotype (Fig. 2c,g). We previously estimated around 100 CENP-A nucleosomes per haplotype in RPE1 cells^40^. Within a ~100-kb centromere domain, this translates to one in five nucleosomes containing CENP-A. Thus, while the loss of H3K9me3 is explained in part by the displacement of histone H3 by CENP-A, our findings suggest that CENP-A seeding also results in the active removal of H3K9 trimethylation.

Canonical alpha-satellite-based centromeres are characterized by distinct dips in 5mCG methylation at regions with high CENP-A occupancy^3,28,41^. Using DiMeLo-seq, we can directly measure endogenous 5mCG on the same single molecules on which we measure exogenous 6mA deposited around H3K9me3 or CENP-A. We found that the neocentromere arose in a broad domain of generally low DNA methylation (Fig. 2b,c,f). Strikingly, the formation of the CENP-A domain induced the removal of the remaining 5mCG methylation, specifically on the CENP-A-containing haplotype (Fig. 2b,c,f). While we cannot directly establish causality, our data on a newly formed neocentromere suggest that CENP-A seeding can specifically drive local CpG demethylation (Fig. 2c–e).

**Extended Data Fig. 1:**
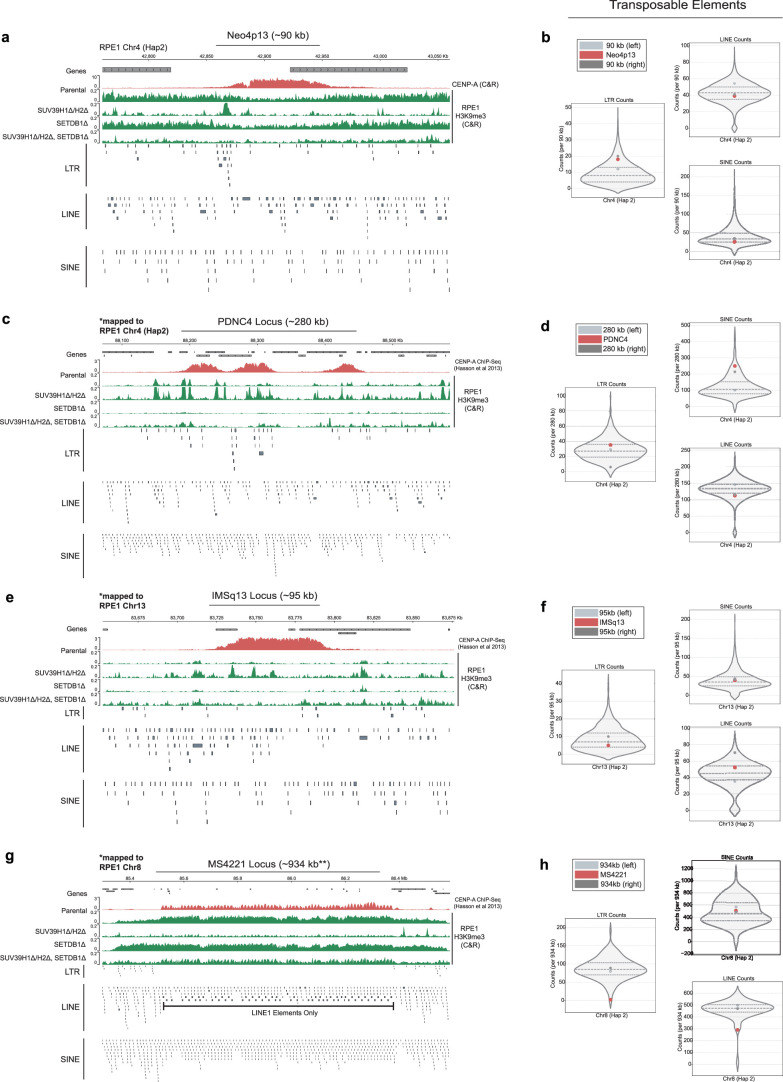
Transposable Element Density and H3K9me3 density at known neocentromere loci. **a, c, e, g**, Retrotransposon density and H3K9me3 CUT&RUN enrichment in RPE1 cells across loci that either feature a neocentromere (Neo4p13) or at loci where CENP-A has been detected in patient-derived neocentromere cells (PDNC4, IMSq13 and MS4221). **b, d, f, h**, Violin plot of total LTR, LINE and SINE density across each neocentromere, highlighting bins directly overlapping with or immediately adjacent to each neocentromere. The bin size for each violin plot is set to the overall CENP-A domain size for the corresponding neocentromere.

Previously, we determined that the overall A+T content of the 4p13 neocentromere region is roughly equal to that of the chromosome average^18^. Here, we analyzed the distribution of transposable elements (TEs) that have previously been implicated in centromere function, although the roles of TEs have been elusive^42^. Using the diploid RPE1 genome^39^, we characterized the genome-wide TE distribution using RepeatMasker (http://www.repeatmasker.org). We found that the 4p13 neocentromere locus features a higher number of long terminal repeats (LTRs) versus the chromosomal averages for Chr4 Hap2 (Extended Data Fig. 1a,b), most of which are present near the telomere-proximal side of the CENP-A peak (Extended Data Fig. 1a). In contrast, long (LINEs) and short (SINEs) interspersed nuclear elements are closer to the genome average (Extended Data Fig. 1a,b).

We also analyzed the loci of previously characterized patient-derived neocentromeres on chromosomes 4, 8 and 13 (PDNC4, MS4221 and IMS13q, respectively)^26,27^. Unlike Neo4p13, the PDNC4 neocentromere at 4q21 is enriched in SINEs, with only a minor increase in LTRs (Extended Data Fig. 1c,d). IMS13q on chromosome 13 showed a slight enrichment for LINEs (Extended Data Fig. 1e,f). Interestingly, at MS4221, although overall depleted in LINEs, CENP-A maps precisely onto LINE1 elements at this neocentromere (Extended Data Fig. 1g,h), consistent with earlier observations^43^. Thus, while interspersed repeats are generally associated with neocentromere activity, there appears to be no unique repeat linked to centromere function. In all cases analyzed, there were numerous other regions across the chromosome that feature higher numbers of all three TE classes (Extended Data Fig. 1), making TEs unlikely to be defining features of neocentromeres. Furthermore, not all neocentromere-forming regions appear to be strongly enriched in broad domains of H3K9me3. Known patient-derived neocentromeres on chromosomes 4q and 13q have appeared in regions that, at least in our RPE genome analysis, are depleted of H3K9me3 or contain more refined H3K9me3 peaks rather than a broad domain of H3K9me3 (Extended Data Fig. 1c,e). These lower levels of H3K9me3 were also observed at different neocentromeres across the 13q arm^27^, as well as at the 4q21 neocentromere, where variation in H3K9m3 levels correlates with changes in CENP-A position^44^.

### Heterochromatin at Neo4p13 forms functional centromere boundaries, maintaining CENP-A domain size

**Fig. 3:**
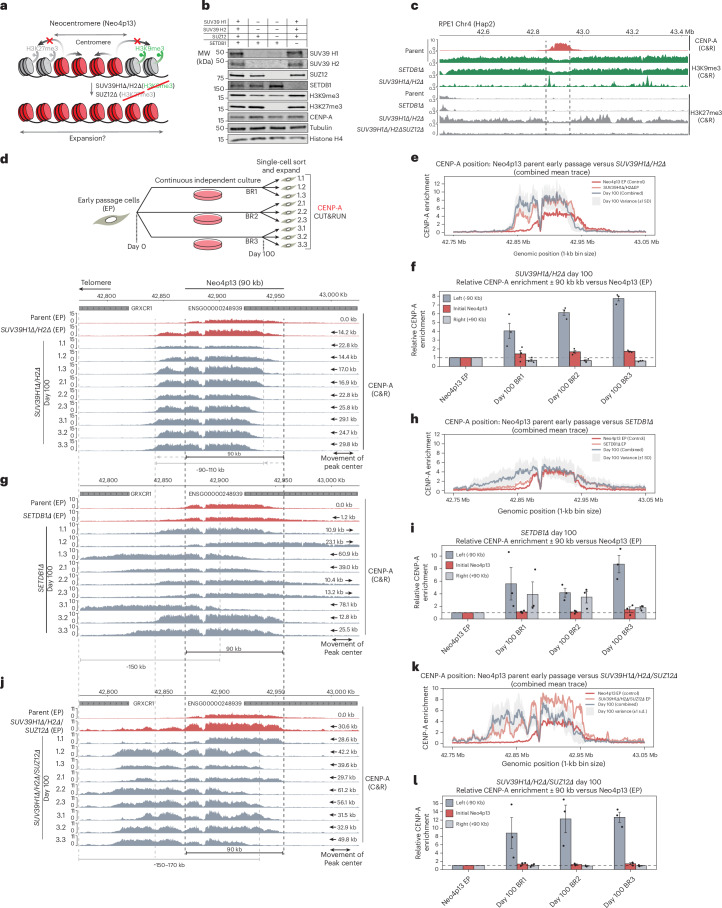
Heterochromatin forms functional boundaries and maintains tight size control of the Neo4p13 locus. **a**, Model showing that heterochromatin acts as a boundary that limits neocentromere expansion and maintains locus size. **b**, Immunoblots for SUV39H1, SUV39H2 and SUZ12 single, double and triple knockouts and SETDB1 knockouts, probed for indicated proteins and histone marks. Histone H4 and α-tubulin were used as loading controls (≥2 independent replicates). **c**, CUT&RUN profiles across the Neo4p13 region for CENP-A, H3K9me3 and H3K27me3 in indicated genotypes; dashed lines mark Neo4p13 centromere boundaries. **d**, CENP-A CUT&RUN comparing early-passage parental Neo4p13 to SUV39H1Δ/H2Δ clones at early passage and after ~100 days; black dotted lines show the initial centromere position and arrows indicate the shift magnitude. **e**, CENP-A enrichment (1-kb bins) across 42.8–43.0 Mb (Chr4 Hap2) for parental (red) versus SUV39H1Δ/H2Δ clones. Early-passage traces are shown in orange and the day 100 mean (BR1–BR3) ± s.d. is shown in gray. **f**, CENP-A enrichment within initial Neo4p13 interval (42.868–42.958 Mb) and flanks 90 kb upstream (‘left’) and downstream (‘right’), for SUV39H1Δ/H2Δ clones normalized to parental early passage (baseline = 1). Data are shown as the mean ± s.e.m. (*n* = 3 biologically replicates). **g**, CENP-A CUT&RUN comparing early-passage parental Neo4p13 to SETDB1Δ clones, analogous to **d**. **h**, CENP-A enrichment analogous to **e** but for parental versus SETDB1Δ clones. **i**, CENP-A enrichment within initial Neo4p13 interval and flanks, analogous to **f** but for SETDB1Δ clones. Data are shown as the mean ± s.e.m. (*n* = 3 biologically replicates). **j**, CENP-A CUT&RUN comparing early-passage parental Neo4p13 to SUV39H1Δ/H2Δ/SUZ12Δ clones, analogous to **d**. **k**, CENP-A enrichment analogous to **e** but for parental versus SUV39H1Δ/H2Δ/SUZ12Δ clones. **l**, CENP-A enrichment within initial Neo4p13 interval and flanks, analogous to **f** but for SUV39H1Δ/H2Δ/SUZ12Δ clones. Data are shown as the mean ± s.e.m. (*n* = 3 biologically replicates). Source data Source data

Given the maintenance of size control of the 4p13 neocentromere that we observed in long-term culture, we hypothesized that adjacent H3K9me3-marked heterochromatin forms an effective boundary (Fig. 3a). We predicted that the H3K9me3 methyltransferases SUV39H1 and SUV39H2 may be responsible for maintaining the heterochromatin domain at 4p13, as these are known to mark pericentric heterochromatin at canonical centromeres^45–47^. We also considered the H3K9me3 methyltransferase SETDB1 as a putative candidate, as this enzyme is known to have roles distinct from SUV39 enzymes, particularly in TE silencing^47–49^.

We constructed *SETDB1*-null (*SETDB1Δ*) and *SUV39H1/H2*-double-null mutant lines (*SUV39H1Δ/H2Δ*). Loss of SUV39H1/H2 resulted in a modest reduction in global H3K9me3 levels, likely because of other H3K9 methyltransferases (Fig. 3b). In contrast, SETDB1 loss did not affect overall H3K9me3 levels detectable by immunoblot (Fig. 3b). Using H3K9me3-directed CUT&RUN, we found that deletion of *SUV39H1/H2* but not SETDB1 resulted in the near-complete loss of H3K9me3 from the neocentromere domain, except for some discrete H3K9me3 peaks (Fig. 3c). While the CENP-A domain maintained its position in SETDB1 mutants (Fig. 3g–i, red tracks), loss of H3K9me3 in *SUV39H1Δ/H2Δ* cells resulted in a substantial drift of the CENP-A domain in a telomere-proximal direction, following the generation and expansion of mutant cells (~50 days in culture) with a telomere-proximal peak center shift of 14.2 kb (Fig. 3d–f, red tracks).

To assess long-term adaptation, we used the clonal culture strategy, outlined in Fig. 1. Following an additional 100 days of continuous proliferation, all *SUV39H1Δ/H2Δ* clones moved to a more telomere-proximal position compared to parental Neo4p13 (Fig. 3d–f, gray tracks), with an average shift of the peak center of 22.6 kb. Moreover, CENP-A enrichment is generally higher across all clones versus parental early-passage cells. Importantly, the overall size of the CENP-A domain remained largely consistent at 90–110 kb in evolved clones, suggesting another layer of size control that maintains neocentromere size, preventing spreading beyond a confined locus.

While deletion of *SETDB1* did not result in notable changes in H3K9me3 and CENP-A positioning initially, analysis of 100-day long-term passaged clones revealed extensive heterogeneity in CENP-A position, in either direction, with a mean shift of peak center of 30.2 kb (Fig. 3g–i, gray tracks). The drift in CENP-A occurred without apparent changes in initial H3K9me3 levels (Fig. 3c). To determine whether SETDB1 possibly has a redundant role in regulating H3K9me3 at the 4p13 neocentromere, we compared H3K9me3 CUT&RUN data in *SUV39H1Δ/H2Δ*, *SETDB1Δ* and combined *SUV39H1Δ/H2Δ/SETDB1Δ* mutant backgrounds (Extended Data Fig. 1a). Upon removal of SUV39H1 and SUV39H12, there remained some more refined H3K9me3 peaks near LTRs within the Neo4p13 region that were removed upon additional *SETDB1* deletion. These findings suggest that SETDB1 has a more subtle role in silencing TE elements in this region and a contributing role in maintaining CENP-A boundaries.

We also analyzed the role of H3K9me3 modifiers in heterochromatin distribution at other RPE1 loci known to be associated with patient-derived neocentromeres (PDNC4, IMSq13 and MS4221)^26^. We found that the discrete H3K9me3 peaks surrounding the PDNC4 and IMSq13 neocentromere loci were largely dependent on SETDB1 (Extended Data Fig. 1c,e). In contrast, H3K9me3 at the MS4221 neocentromere locus appeared more broadly SUV39-regulated (Extended Data Fig. 1g). Thus, neocentromere loci appear to be at least in part influenced by SETDB1 and/or SUV39H1/H2 activity.

Loss of H3K9me3 may result in compensatory modes of heterochromatinization. Indeed, we observed an increase in Polycomb repressive complex 2 (PRC2)-mediated H3K27me3 facultative heterochromatin upon loss of SUV39-mediated H3K9me3 at 4p13 (Fig. 3c and Extended Data Fig. 5a), consistent with previous studies^29^, a phenomenon also observed on H3K9me3-depleted human artificial chromosomes^50^. To remove H3K27me3, we disrupted the gene encoding suppressor of zeste 12 (SUZ12), an essential component of the PRC2 complex (Fig. 3b)^51^. Deletion of *SUZ12* resulted in a complete loss of H3K27me3 in *SUV39H1Δ/H2Δ/SUZ12Δ* triple-mutant Neo4p13 cells (Fig. 3b,c). Strikingly, size control of the CENP-A domain is largely lost in this context, resulting in a doubling in size compared to the original Neo4p13 locus (Fig. 3j–l). CENP-A moves much further than in *SUV39H1Δ/H2Δ* to a size up to 150 kb and breaching into the nearest gene boundary (GRXCR1), with a mean peak center shift of 41.3 kb. Combined, these results indicate that heterochromatin marked by H3K9me3 and H3K27me3 can form clear functional boundaries at this neocentromere and maintain tight size control of the CENP-A domain, independent of 5mCG methylation and alpha-satellites.

### Canonical alpha-satellite-based centromeres are characterized by heterochromatin dips

**Fig. 4:**
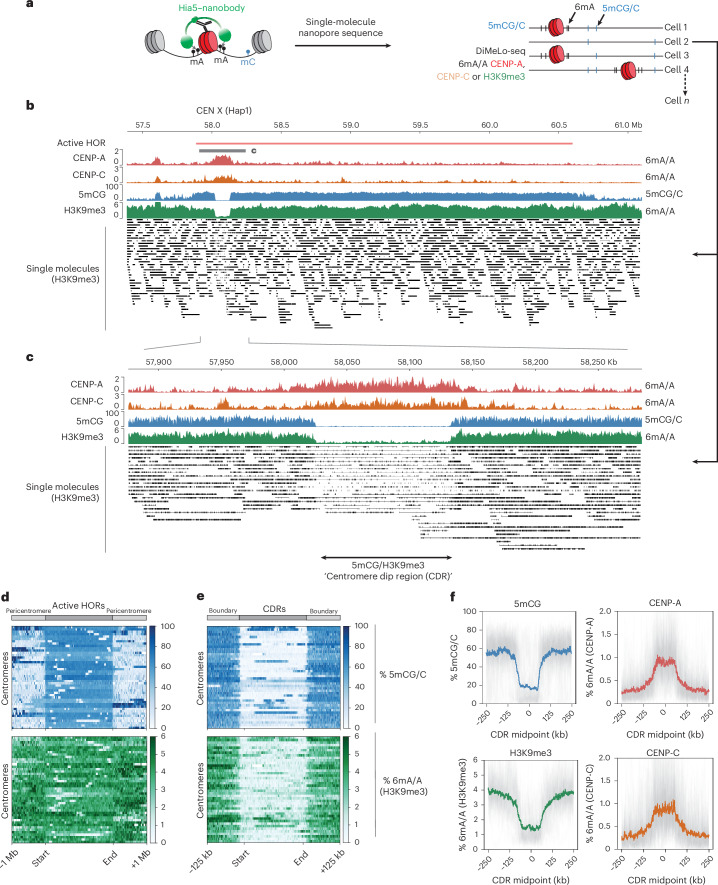
Heterochromatin dips are characteristic of canonical centromeres. **a**, Diagram of DiMeLo-Seq workflow with averaged 6mA/A probabilities and single-molecule alignments. Vertical lines of each read represent exogenous 6mA directed toward CENP-A, CENP-C or H3K9me3. **b**, View of the entire HOR of CENX (Hap1), with DiMelo-Seq enrichments for CENP-A (red), CENP-C (orange) and H3K9me3 (green), as well as 5mCG enrichment (blue). Single molecules of each long read for H3K9me3 (black). Specifically where CENP-A and CENP-C reside, there is the characteristic dip in DNA methylation (5mCG) and H3K9me3 heterochromatin. **c**, A closer view of CENX, showing the dip in H3K9me3 and boundaries surrounding the active CENP-A/CENP-C-containing region. Single molecules at this site contain largely unmethylated adenine-called reads. **d**, Heat maps illustrating the distribution of 5mCG (top, blue) and H3K9me3-directed 6mA (bottom, green) modifications across centromeric active HOR regions. Each line on the heat map spans the active HOR of a single centromere and flanking regions ±1 Mb upstream and downstream. All active HOR regions were scaled to the same length to allow direct comparison of methylation patterns across all centromeres. Signal intensity is represented as the mean methylated fraction across fixed genomic bins, with darker shades indicating higher modification levels. The start and end of the HOR regions are indicated, highlighting methylation patterns in and around these active centromeric domains. **e**, Heat maps as in **d** but specifically across CDRs, as defined by the boundary of 5mCG dips on either side of each CENP-A-containing region. All CDRs were scaled to the same length. **f**, Individual traces for each centromere (gray) and aggregated (colored) modification traces of CENP-A, CENP-C, H3K9me3 and 5mCG across all CDRs, centered on CDR midpoints, with ±250-kb flanking regions.

Next, we investigated whether similar boundary mechanisms exist at canonical centromeres. Characteristic features of canonical centromeres, such as repetitive alpha-satellite DNA and high levels of DNA methylation, may also contribute to maintaining CENP-A positioning. To assess chromatin organization at highly repetitive satellite DNA, we used ONT ultralong-read DiMelo-seq in combination with the isogenomic RPE reference genome^39^ to accurately map CENP-A, CENP-C and H3K9me3 occupancy at canonical centromeres (Fig. 4a)^37^. We performed this analysis in the same RPE1-Neo4p13-bearing cell line for consistency across our experiments. As ONT enables direct genomic DNA sequencing lacking PCR amplification, we can determine the single-molecule occupancy of our target of interest across centromeres or pericentromeres.

**Extended Data Fig. 2:**
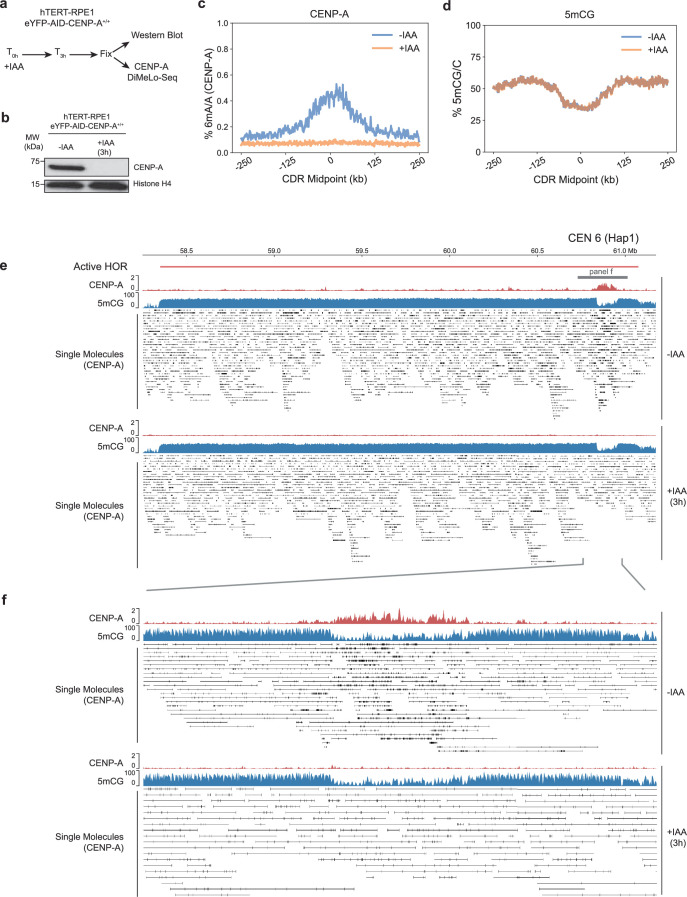
On-target specificity of CENP-A-DiMelo-seq. **a**, Schematic of 3 hr auxin depletion using hTERT-RPE1 eYFP-AID-CENP-A^+/+^ cells^52^. **b**, Immunoblot for CENP-A and Histone H4 on nuclear extracts +/- 3hr IAA treatment (Representative of n = 2 independent experiments). **c**, CENP-A-directed 6mA/A plot with the mean 6mA trace for +/- IAA conditions for all centromeres, overlayed for comparison. **d**, Combined 5mCG/C plot with the mean 6mCG trace for +/- IAA conditions for all centromeres, overlayed for comparison. **e**, View of the entire HOR of CEN6 (Hap1) +/- IAA conditions with CENP-A 6mA/A and 5mCG/C averaged genomic tracks, and single molecule alignments with CENP-A-directed 6mA modifications. **f**, Zoom into the CDR of CEN6 (Hap1) +/- IAA conditions with CENP-A 6mA/A and 5mCG/C averaged genomic tracks, and single molecule alignments with CENP-A-directed 6mA modifications. Source data

To determine the specificity of the CENP-A DiMelo-seq 6mA signal, we used an endogenous homozygous knock-in AID-eYFP-CENP-A hTERT-RPE1 cell line to auxin-deplete all endogenous CENP-A^52^, followed by CENP-A DiMeLo-seq (Extended Data Fig. 2a). CENP-A was effectively depleted from cells (Extended Data Fig. 2b) and the corresponding CENP-A 6mA signal was specifically eliminated from all CDRs, indicating that 6mA is CENP-A specific and providing us with a true background CENP-A 6mA/A signal (Extended Data Fig. 2c,e,f). Consistent with previous observations^3,28,53^, active alpha-satellite HOR arrays generally contained high levels of DNA methylation (Fig. 4b–f, blue). Moreover, active centromeric regions containing CENP-A featured the previously characterized CDRs, depleted of 5mCG, which tightly inversely correlate with all CENP-A (red) and CENP-C (orange) containing regions (Fig. 4b,c,f). Interestingly, 5mCG levels were unaffected by acute loss of CENP-A (Extended Data Fig. 2d–f), indicating that the local dip in DNA methylation is relatively stable, at least within a few hours of CENP-A depletion.

**Extended Data Fig. 3:**
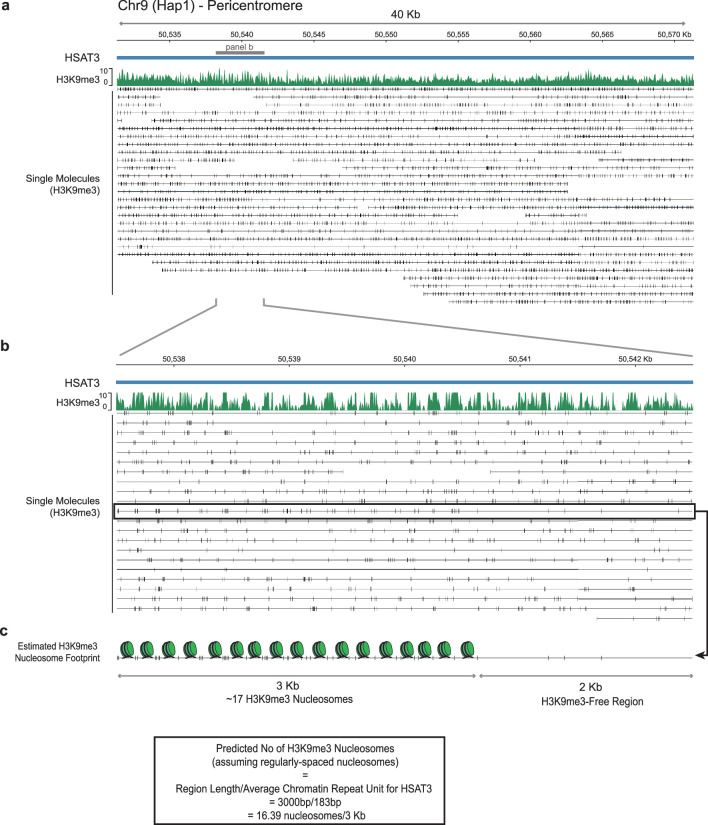
Nucleosome-level sensitivity of H3K9me3 DiMelo-seq at constitutive pericentromeric heterochromatin. **a**, Visualisation of a 40 kb region on human satellite 3 (HSat3) of chromosome 9, showing H3K9me3 averaged m6A/A track (green) and single molecule alignments (black, bottom). Black vertical lines represent H3K9me3-directed 6mA modification. **b**, Zoom in to a 5 kb region of HSat3 showing H3K9me3 averaged m6A/A track (green) and single molecule alignments (black, bottom). 6mA modifications appear as regularly spaced nucleosome-like intervals. **c**, Estimation of H3K9me3 nucleosome footprinting on a 5kb region of HSat3, showing a 3 kb H3K9me3-dense and a 2 kb H3K9me3-free region. On this single molecule, we can estimate nucleosome positioning of H3K9me3-containing nucleosomes to be approximately 17 nucleosomes within this 3kb H3K9me3-dense region (176 bp/nucleosome, which is consistent with previous estimates of 183 bp/nucleosome for HSat3 heterochromatic regions based on Fiber-Seq measurements^57^.

H3K9me3 is normally present in high densities in pericentric regions in classical constitutive HP1-mediated heterochromatin domains^54–56^. To determine the resolution of our H3K9me3 DiMeLo-seq, we first assessed H3K9me3 in pericentric regions, specifically using HSat3 as an example (Extended Data Fig. 3a). We could achieve near-single-nucleosome resolution for H3K9me3, giving us an estimate of methylated nucleosome densities in these regions that is consistent with the reported average chromatin repeat unit length for HSat3 (Extended Data Fig. 3b,c)^57^.

**Extended Data Fig. 4:**
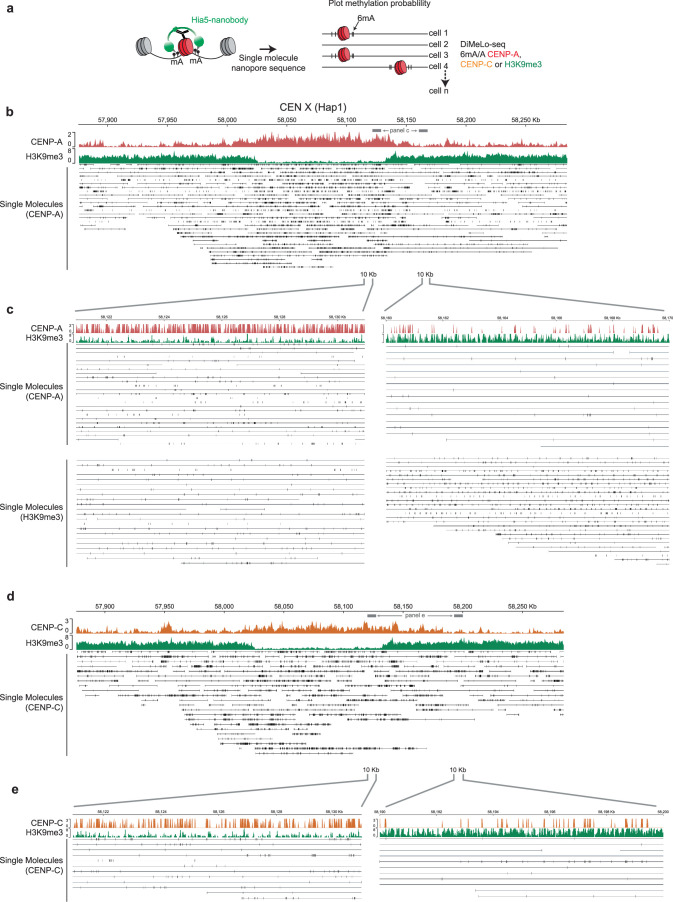
Nucleosome-level view of CENP-A, CENP-C and H3K9me3 DiMelo-seq at centromere boundary. **a**, Schematic of DiMelo-Seq workflow and visualisation of single molecule alignment. Black vertical lines represent 6mA modification. **b**, Centromere X (Haplotype 1) visualised with CENP-A (red, 6mA/A) and H3K9me3 (green, 6mA/A). Single-molecule alignments with 6mA modifications for CENP-A (bottom), show the specificity of the CENP-A signal to the CDR. **c**, 10kb zoom in to regions inside (left) versus outside (right) of the CENX CDR for single DNA molecules, showing the specificity for CENP-A and H3K9me3 detection at our chosen m6A probability filtering. **d**, Centromere X (Haplotype 1) visualised with CENP-C (orange, 6mA/A) and H3K9me3 (green, 6mA/A). Single-molecule alignments with 6mA modifications for CENP-C (bottom), show the specificity of the CENP-A signal to the CDR. **e**, 10kb zoom in to regions inside (left) versus outside (right) of the CENX CDR for single DNA molecules, showing the specificity for CENP-C detection at our chosen m6A probability filtering.

In the classic centric–pericentric dichotomy, heterochromatin, marked by H3K9me3, is considered to be largely pericentric, whereas the kinetochore-forming CENP-A domain is largely depleted of heterochromatin^29,30,35,58^. Moreover, centromeres on human artificial chromosomes resist silencing mediated by H3K3me3 and H3K27me3 (ref. ^50^) and H3K9me3 is not required for CENP-A-driven epigenetic memory^59^, suggesting an inverse relationship and functional boundary potential. However, using DiMeLo-seq, it was unambiguously shown that H3K9me3 is also present within HORs that form a template for CENP-A^37^. Consistent with these data, we found high levels of H3K9me3 across all pericentromeres and active HORs in RPE1 (Fig. 4b,c,d, green). Importantly, we identified a distinct depletion in H3K9me3 within the CENP-A domain at all centromeres, forming a tight boundary around the CENP-A/CENP-C domain in a manner analogous to 5mCG (Fig. 4c,e,f and Extended Data Fig. 4). Thus, the CDR is defined not only by depletion of 5mCG but also by a dip in the heterochromatin mark H3K9me3.

### Centromeric H3K9me3 is differentially controlled by SETDB1, SUV39 and noncanonical SUZ12 functions

**Fig. 5:**
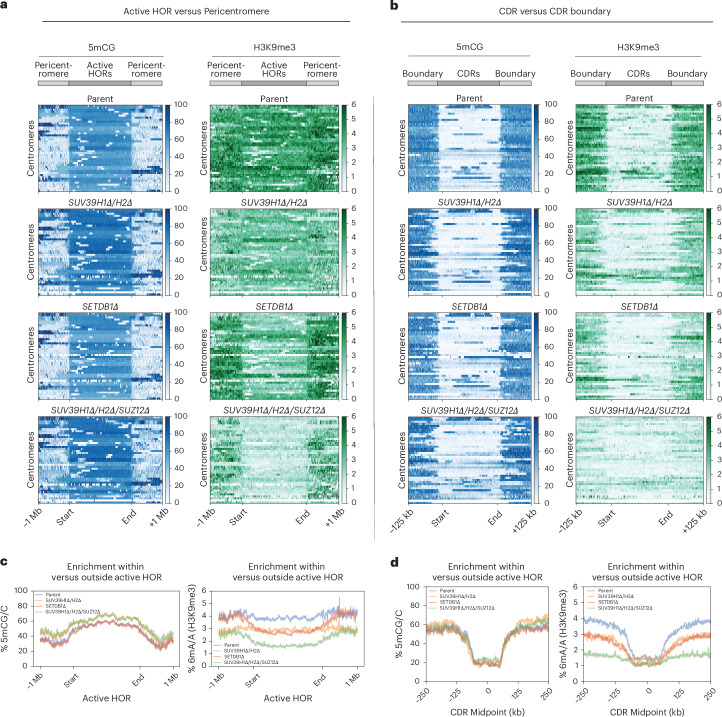
Centromeric H3K9me3 is regulated by a combination of SETDB1, SUV39 and noncanonical SUZ12 activities. **a**, Heat maps for WT, *SUV39H1Δ/H2Δ*, *SETDB1Δ* and *SUV39H1Δ/H2Δ/SUZ12Δ* cell lines, illustrating the distribution of 5mCG (blue) and H3K9me3-directed 6mA (green) modifications across centromeric active HORs. Each line on the heat map spans the active HORs of a single centromere and flanking regions ± 1 Mb upstream and downstream. All active HOR regions were scaled to the same length to allow direct comparison of methylation patterns across all centromeres. **b**, Heat maps as in **a** but specifically across CDRs, as defined by the boundary of 5mCG dips on either side of each CENP-A-containing region. All CDRs were scaled to the same length. **c**, Average DNA methylation signal left; (in % 5mCG/C) and H3K9me3 signal (right; in % 6mA/A), across all active HOR regions for indicated mutants (scaled equally), flanked by ±1 Mb. **d**, Average DNA methylation signal (left; in % 5mCG/C) and H3K9me3 signal (right; in % 6mA/A) for indicated mutants, across all CDRs (scaled equally) regions flanked by ±250 kb.

To uncover the importance of heterochromatin boundaries at canonical alpha-satellite-based centromeres, we assessed H3K9me3 levels in the *SETDB1Δ*, *SUV39H1Δ/H2Δ* and *SUV39H1Δ/H2Δ/SUZ12Δ* double and triple mutants described above. We found that, in *SUV39H1Δ/H2Δ* double mutants, H3K9me3 levels were decreased both across the flanking pericentromere and within centromeric active HORs, compared to parental cells (Fig. 5a,c). Surprisingly, we found that SETDB1 contributes to H3K9me3 deposition specifically within the centromeric HORs and not in the flanking pericentric regions (Fig. 5a,c), indicating it has centromere-specific roles distinct from SUV39 enzymes.

*SUV39H1Δ/H2Δ/SUZ12Δ* triple mutants offered the most prominent effect on H3K9me3. Whilst levels were reduced in the pericentromeric regions to similar levels as in *SUV39H1Δ/H2Δ* double-mutant cells, there was a striking and substantial loss of H3K9me3 that specifically affected active HORs, even beyond that observed in *SETDB1Δ* cells (Fig. 5a,c). Hence, whilst loss of *SUV39H1* and *SUV39H2* generally reduced H3K9me3 in pericentric and alpha-satellite-containing chromatin, *SETDB1* and *SUZ12* deletion appeared to have selective effects on alpha-satellite H3K9me3.

**Extended Data Fig. 5:**
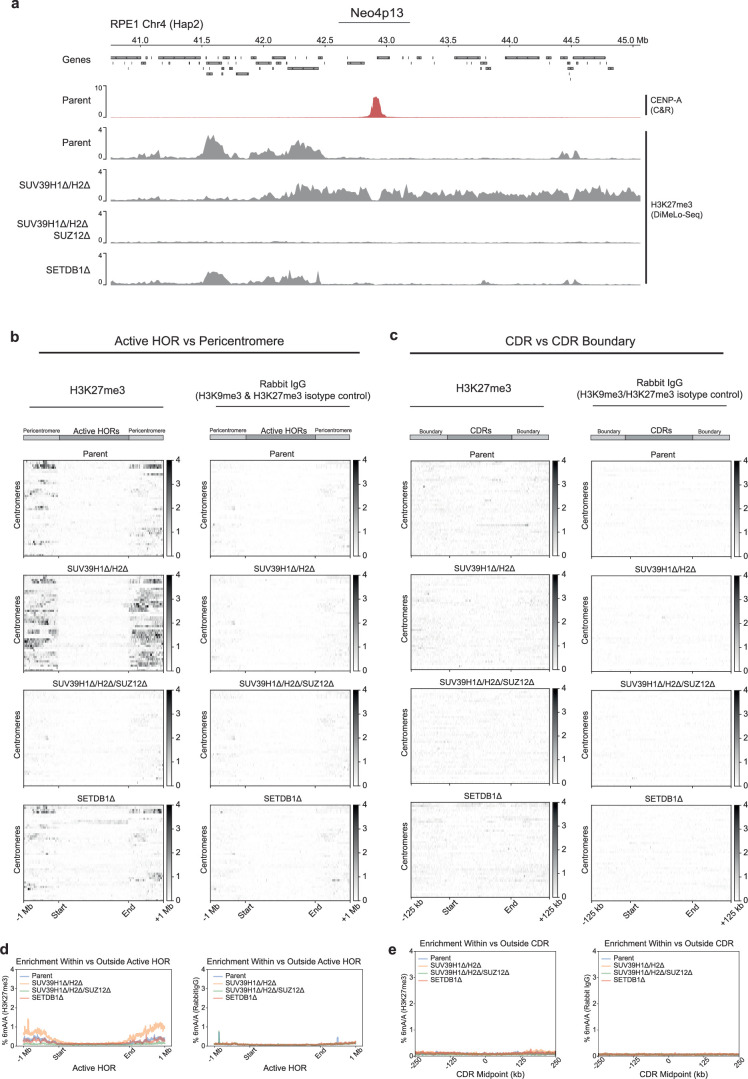
Characterisation of H3K27me3 DiMelo-Seq at Neo4p13 and Canonical Centromeres. **a**, Visualisation of the broader Neo4p13 region (40.75 Mb to 45.0 Mb) showing CENP-A (red) and H3K27me3 averaged m6A/A track (grey) for each of parental (RPE-Neo4p13) and *SUV39H1Δ/H2Δ*, *SUV39H1Δ/H2Δ/SUZ12Δ* and *SETDB1Δ*. **b**, Heatmaps for Parental, *SUV39H1Δ/H2Δ*, *SUV39H1Δ/H2Δ/SUZ12Δ* and *SETDBΔ* cell lines, illustrating the distribution of H3K27me3-directed 6mA (left) and Rabbit IgG-directed 6mA (right) modifications across centromeric Active HORs. Each line on the heatmap spans the Active HORs of a single centromere and flanking regions ±1 Mb upstream and downstream. All Active HOR regions have been scaled to the same length to allow direct comparison of methylation patterns across all centromeres. **c**, Heatmaps for H3K27me3 and Rabbit IgG as in (**b**), but specifically across Centromere Dip Regions (CDRs), as defined by the boundary of 5mCG dips on either side of each CENP-A-containing region. All CDRs have been scaled to the same length. **d**, Average 6mA 6mA modification signal for H3K27me3 (left) and Rabbit IgG (right) across all Active HOR regions (scaled equally), flanked by ±1 Mb. **e**, Average indicated 6mA modification signal of H3K27me3 (left) and Rabbit IgG (right) across all CDRs regions flanked by ±250 kb.

Next, we determined whether loss of H3K9me3 in *SUV39H1Δ/H2Δ* and *SETDB1Δ* mutants resulted in compensation by H3K27me3, assessed by DiMeLo-seq. We found no strong enrichment for the PRC2-mediated mark within the HOR in parental active HORs (Extended Data Fig. 5b,c, top). Loss of H3K9me3 in *SUV39H1Δ/H2Δ* and *SETDB1Δ* mutants did not result in any compensatory H3K27me3 deposition, except for pericentric, divergent satellites in *SUV39H1Δ/H2Δ* (Extended Data Fig. 5b–e). These observations indicate that the contributing role of SUZ12 to maintaining H3K9m3 in *SUV39H1Δ/H2Δ* mutants is not mediated by H3K27me3 but possibly through a direct noncanonical contribution of this PRC2 subunit to heterochromatin maintenance.

We then analyzed the effect of heterochromatin mutants on H3K9me3 boundaries at the CENP-A-containing DNA methylation-depleted regions (CDRs). We found local H3K9me3 boundaries to be effectively eliminated in *SUV39H1Δ/H2Δ/SUZ12Δ* backgrounds, with visible reductions in boundary strength also apparent in *SUV39H1Δ/H2Δ* and *SETDB1Δ* (Fig. 5b,d). Importantly, the 5mCG-defined CDRs were largely maintained (Fig. 5b,d). Therefore, in this context, we primarily assessed the contributions of H3K9me3 to centromere position, largely independent of the role of DNA methylation or H3K27me3 chromatin.

### Heterochromatin is the primary functional CENP-A boundary at canonical centromeres

**Fig. 6:**
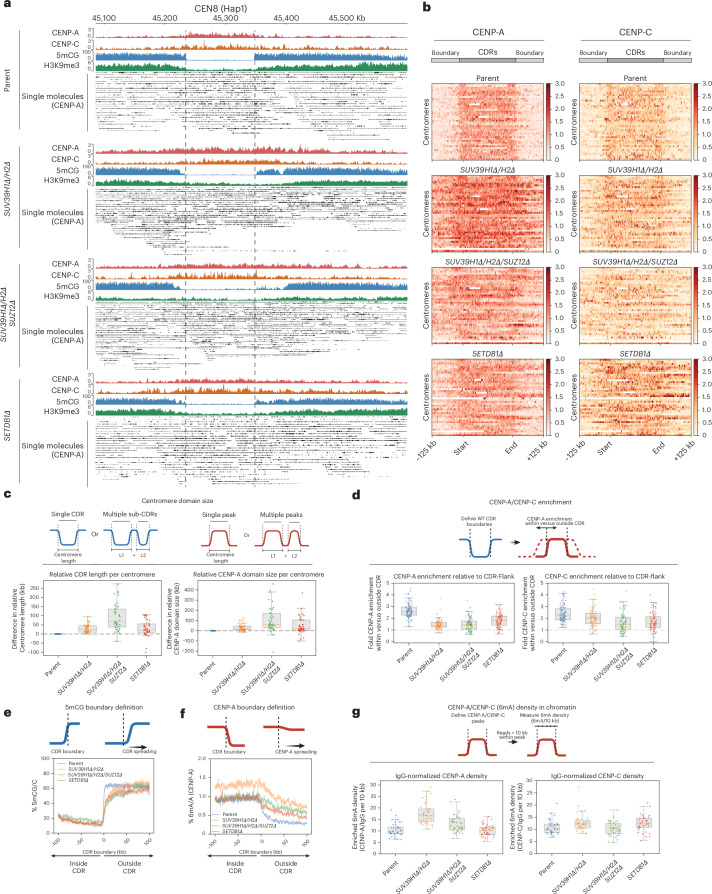
Heterochromatin forms a functional boundary at canonical centromeres. **a**, Whole-HOR view of CEN8 (Hap1) in parental (RPE-Neo4p13), *SUV39H1Δ*/*H2Δ*, *SETDB1Δ* and *SUV39H1Δ/H2Δ/SUZ12Δ* lines showing aggregated DiMeLo-seq for CENP-A (red), CENP-C (orange), 5mCG (blue) and H3K9me3 (green) plus single-molecule alignments of CENP-A–directed 6mA (black). **b**, Heat maps of CENP-A and CENP-C across CDRs, defined by 5mCG dips flanking each parental CENP-A region. Each row is one CDR or centromere; CDRs are numerically sorted and scaled by length. **c**, Centromere domain size. Left, total CDR/sub-CDR length relative to the corresponding parental centromere. Right, CENP-A domain length relative to parental. Box plots summarize parent and mutants with individual centromeres as points. The dashed line denotes the parental and the orange line denotes the median (one BR). **d**, CENP-A/CENP-C enrichment. Ratio of CENP-A signal within CDRs versus flanks (±100 kb). The dashed line denotes equal inside and outside and the orange line denotes the median (from one BR). **e**, 5mCG boundary definition. All 5mCG CDR boundary profiles are centered on boundaries (±100 kb); the mean aggregated 5mCG is shown for each sample. **f**, CENP-A boundary definition. CENP-A enrichment inside versus outside the parental 5mCG-defined CDR boundary (±100 kb); the mean aggregated CENP-A (6mA/A) trace is shown for each sample. **g**, CENP-A/CENP-C 6mA density. Box plots of 6mA enrichment density per 10 kb for reads within CENP-A peaks, normalized to matched IgG controls (one BR). For box plots in **c**,**d**,**g**, the center line is the median, the box denotes the 25th–75th percentiles, whiskers show the minimum and maximum within 1.5× the IQR and data points represent individual centromeres. Source data

Next, having established means to selectively deplete heterochromatin from active HORs and pericentric HSats, we assessed its contribution to CENP-A positioning. In parental cells, we observed a tight positioning of CENP-A and CENP-C within the CDR, with levels rapidly dropping off outside of the 5mCG CDR boundaries (Figs. 4f and 6a,b,f). In *SETDB1Δ*, *SUV39H1Δ/H2Δ* and *SUV39H1Δ/H2Δ/SUZ12Δ* mutants, we observed a notable overall expansion of CENP-A following ~50 days in culture (time to generate these knockouts) (Fig. 6a–d,f). The majority of centromeres moved beyond their original CDR boundaries of parental cells, most prominently in the *SUV39H1Δ/H2Δ/SUZ12Δ* triple-mutant cells, where H3K9me3 levels were the most reduced. The CENP-A enrichment ratio within versus outside the CDR in control samples dropped in both mutants (Fig. 6d), even below a ratio of 1 in many centromeres, indicating that CENP-A became more enriched in the surrounding 100-kb region than within the originally defined centromere boundaries.

**Extended Data Fig. 6:**
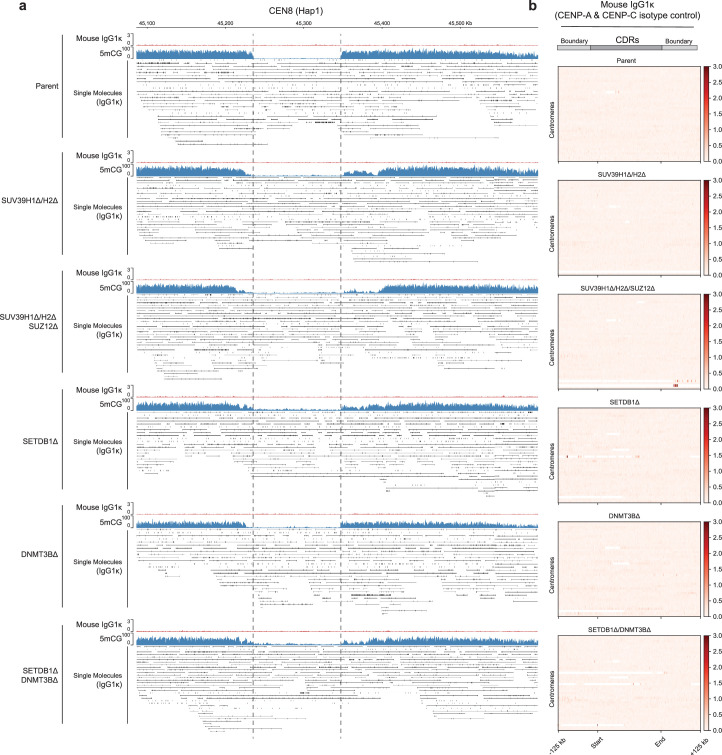
Mouse IgG1 isotype control enrichment at centromeres. **a**, Overview of the entire HOR of CEN 8 (Hap 1), as per Fig. 6, for Parental (RPE-Neo4p13), *SUV39H1Δ/H2Δ*, *SUV39H1Δ/H2Δ/SUZ12Δ*, *SETDB1Δ*, *DNMT3BΔ* and *SETDB1Δ/DNMT3BΔ* cell lines with aggregate DiMelo-Seq enrichments for Mouse IgG1 (red) and 5mCG enrichment (blue). Single molecules alignments for CENP-A-directed 6mA (black). **b**, Heatmaps for each mutant illustrating the distribution of Mouse IgG1 (red) across Centromere Dip Regions (CDRs), as defined by the boundary of 5mCG dips on either side of each CENP-A-containing region in parental RPE-Neo4p13. Each line on the heatmap represents a single CDR/Centromere. All CDRs have been sorted numerically and scaled to the same length for comparison.

Quantitative analysis of 6mA read density normalized to IgG (Extended Data Fig. 6) across the HORs revealed that CENP-A and CENP-C density was largely maintained within CENP-A peaks in *SETDB1Δ* and increased in *SUV39H1Δ/H2Δ* conditions (Fig. 6g). Notably, CENP-A and CENP-C densities did not fall below parental density, indicating that the total copy number of CENP-A nucleosomes (and, to a lesser extent, CENP-C) across HORs was increased rather than diluted across the expanded CDRs (Fig. 6g). Interestingly, the DNA methylation dips were largely maintained, even if H3K9me3 levels were uniformly low across the CDR and adjacent HORs (Figs. 5d and 6a,e). Nevertheless, we could observe erosion of 5mCG at the centromere boundaries, with a mean increase in CDR size of 18% (*SETDB1Δ*), 19% (*SUV39H1Δ/H2Δ*) and 56% (*SUV39H1Δ/H2Δ/SUZ12Δ*), indicating that the H3K9me3-depositing machinery had an indirect effect on DNA methylation (Fig. 6e). In summary, the expansion of CENP-A correlates with the loss of H3K9me3 and, to a lesser extent, with a reduction in DNA methylation at the boundary. These observations indicate that H3K9me3 heterochromatin is the primary functional boundary maintaining CENP-A position.

### DNMT3B contributes to 5mC boundaries and CENP-A positioning

**Extended Data Fig. 7:**
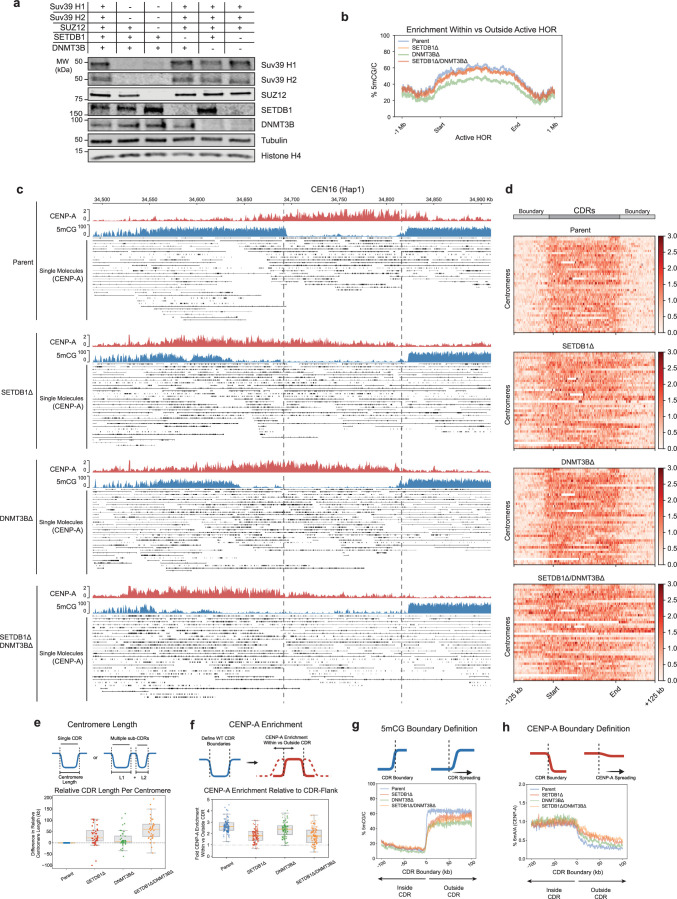
Loss of DNMT3B leads to minor CENP-A repositioning. **a**, Extended immunoblots as shown in Fig. 3b, now including *DNMT3BΔ* and *SETDB1Δ/DNMT3BΔ* characterisation (Representative of n≥2 independent replicates). **b**, Average 5mCG/C modification signal across all Active HOR regions (scaled equally), flanked by ±1000 Kb. **c**, Overview of the entire HOR of CEN 16 (Hap 1) for Parental (RPE-Neo4p13), *SETDB1Δ*, *DNMT3BΔ* and *SETDB1Δ/DNMT3BΔ* cell lines with aggregate DiMelo-Seq enrichments for CENP-A (red) and 5mCG enrichment (blue). Single-molecule alignments for CENP-A-directed 6mA (black). **d**, Heatmaps for each mutant illustrating the distribution of CENP-A (red) across Centromere Dip Regions (CDRs), as defined by the boundary of 5mCG dips on either side of each CENP-A-containing region in parental cells. Each line on the heatmap represents a single CDR/Centromere. All CDRs have been sorted numerically and scaled to the same length for comparison. **e, Centromere length** as defined by the difference in sum total length of CDR/sub-CDRs relative to the corresponding parent centromere. From one biological replicate. **f, CENP-A Enrichment:** Box and strip plot depicting the ratio of CENP-A enrichment within CDRs relative to their flanking regions for parent and mutant lines. A dashed reference line at 1 indicates equal enrichment within and outside the HORs. From one biological replicate. For boxplots in **e** and **f** above: boxplot centre=median; box bounds=25th/75th percentiles; whiskers=min/max within 1.5×IQR from box edges. Datapoints=individual centromeres. **g, 5mCG boundary definition:** plot of all 5mCG CDR boundary profiles centred on CDR boundaries (±100 Kb). Mean of combined aggregated traces is shown for all samples. **h, CENP-A boundary definition:** Plot of CENP-A enrichment inside versus outside the CDR boundary (defined by Parental 5mCG) for each cell line (±100 Kb). Mean of combined CENP-A (6mA/A) aggregated trace is shown for all samples. Source data

We find that, in heterochromatin mutants, the dip in DNA methylation (CDR) is largely maintained (Fig. 5b). Nevertheless, the tight correlation between the CENP-A domain and reduced DNA methylation^3,28,41^ suggest a functional link. Indeed, recent experiments directly implicated CpG methylation in maintaining CENP-A levels and boundaries^60^. The de novo DNA methyltransferase DNMT3B is associated with centromeric and pericentromeric components in various contexts, including CENP-C^61^, Mis18α (ref. ^62^), HP1α (ref. ^45^) and SUV39H1 (ref. ^45^). We deleted *DNMT3B* in parental and *SETDB1Δ* backgrounds to assess any individual or combinatorial contributions toward CENP-A position (Extended Data Fig. 7a). Whilst *DNMT3BΔ* generally reduced 5mCG methylation in the active HOR by 26% (Extended Data Fig. 7b), CENP-A position remained relatively constant at the vast majority of centromeres (Extended Data Fig. 7d,f). The removal of *DNMT3B* in the context of a *SETDB1* mutant in which heterochromatin boundaries are eroded resulted in a more notable loss of CENP-A domain definition (Extended Data Fig. 7c,d,h). For instance, in the absence of *SETDB1*, loss of *DNMT3B* led to CENP-A-invaded monomeric satellite regions flanking the active HOR at CEN16 Hap1 (characterized by non-5mCG-methylated adjacent satellites) (Extended Data Fig. 7c,e,g). These results indicate a role for DNMT3B in maintaining DNA methylation, particularly when heterochromatin boundaries are defective.

### CENP-A drift at canonical centromeres over time is exacerbated by heterochromatin defects

**Extended Data Fig. 8:**
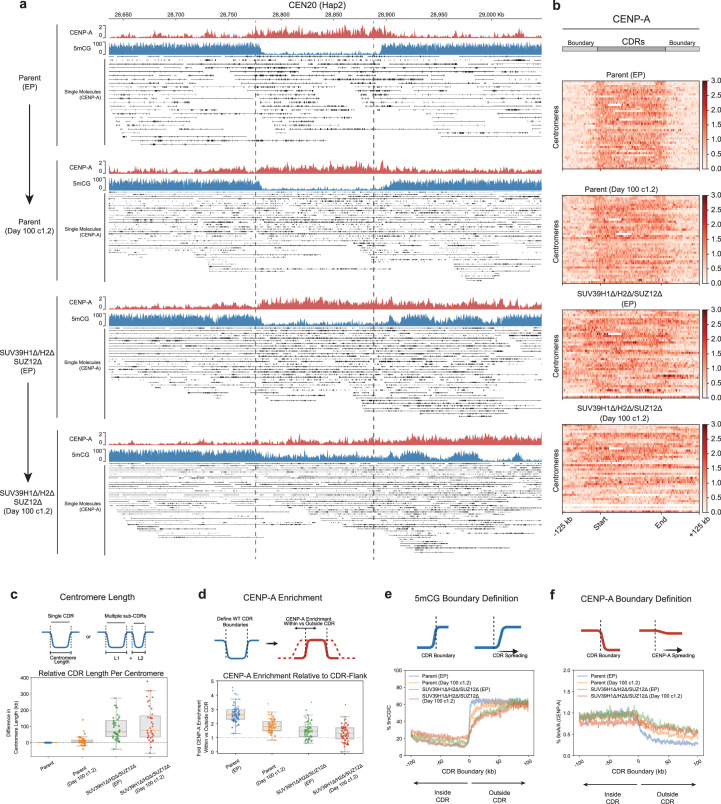
Canonical centromere drift over time, restricted by heterochromatin. **a**, View of the entire HOR of CEN 20 (Hap 2) for Parental (RPE-Neo4p13), Parental (Day 100 Cone 1.2), *SUV39H1Δ/H2Δ/SUZ12Δ* (early Passage, EP) and *SUV39H1Δ/H2Δ/SUZ12Δ* (Day 100 Clone 1.2) with aggregate DiMelo-Seq enrichments for CENP-A (red) and 5mCG enrichment (blue). Single molecules alignments for CENP-A-directed 6mA (black). **b**, Heatmaps for each mutant illustrating the distribution of CENP-A (red) across Centromere Dip Regions (CDRs), as defined by the boundary of 5mCG dips on either side of each CENP-A-containing region in parental RPE-Neo4p13. Each line on the heatmap represents a single CDR/Centromere. All CDRs have been sorted numerically and scaled to the same length for comparison. **c, Centromere length** as defined by the difference in sum total length of CDR/sub-CDRs relative to the corresponding parent centromere. A dashed reference line at 1 indicates early passage parental centromere length. From one biological replicate. **d, CENP-A Enrichment:** Box plot depicting the ratio of CENP-A enrichment within CDRs relative to their flanking regions (±100 Kb) for parent and mutant lines. From one biological replicate. For boxplots in **c** and **d** above: boxplot centre=median; box bounds=25th/75th percentiles; whiskers=min/max within 1.5×IQR from box edges. Datapoints=individual centromeres. **e, 5mCG boundary definition:** plot of all 5mCG CDR boundary profiles centred on CDR boundaries (±100 Kb). Mean of combined aggregated traces is shown for all samples. **f, CENP-A boundary definition:** Plot of CENP-A enrichment inside versus outside the CDR boundary (defined by Parental 5mCG) for each cell line (±100 Kb). Mean of combined CENP-A (6mA/A) aggregated trace is shown for all samples.

Given our observations that Neo4p13 drifts gradually over time (Fig. 1d), we sought to determine whether canonical centromeres display a similar behavior. We picked a parental clone (clone 1.2; Fig. 1d) and *SUV39H1Δ/H2Δ/SUZ12Δ* clone (clone 1.2; Fig. 3j) in which the CENP-A domain at the Neo4p13 locus shifted substantially following 100 days of proliferation. We then performed CENP-A DiMelo-seq to determine any differences in CENP-A position and size over time. Similar to the neocentromere, canonical centromeres move subtly with time over the 100-day period (Extended Data Fig. 8). Parental centromere length (Extended Data Fig. 8c) and CDR boundary definition (Extended Data Fig. 8e) were largely maintained or subtly eroded after 100 days. CENP-A position began to gradually shift from the initial centromere boundary (Extended Data Fig. 8d,f). In contrast, already in early-passage *SUV39H1Δ/H2Δ/SUZ12Δ* cells and in day 100 clones, the CENP-A domain shifted substantially beyond any drift observed in parental cells, even following 100 days of proliferation (Extended Data Fig. 8d,f). In addition, centromere length expanded (Extended Data Fig. 8c) and CDR definition was greatly eroded (Extended Data Fig. 8e) in these clones. We also observed cases where CENP-A position almost entirely vacated the parental position in the *SUV39H1Δ/H2Δ/SUZ12Δ* 100-day background (Extended Data Fig. 8a,b). Interestingly, here, 5mCG methylation began to reoccupy the initial CDR in the absence of CENP-A (Extended Data Fig. 8a, bottom). This suggests that, in addition to our observation at the neocentromere, where seeding CENP-A coincides with a loss of DNA methylation, the inverse can also happen, where the gradual loss of CENP-A results in the reestablishment of DNA methylation. Moreover, our assessment of long-term CENP-A domain dynamics indicates that the dramatic movement of CENP-A in methyltransferase mutants is the result of the loss of these enzymes and not simply a consequence of clonal selection and long-term culture.

### Loss of heterochromatin within HORs results in new CDRs and ectopic functional CENP-A domains

**Fig. 7:**
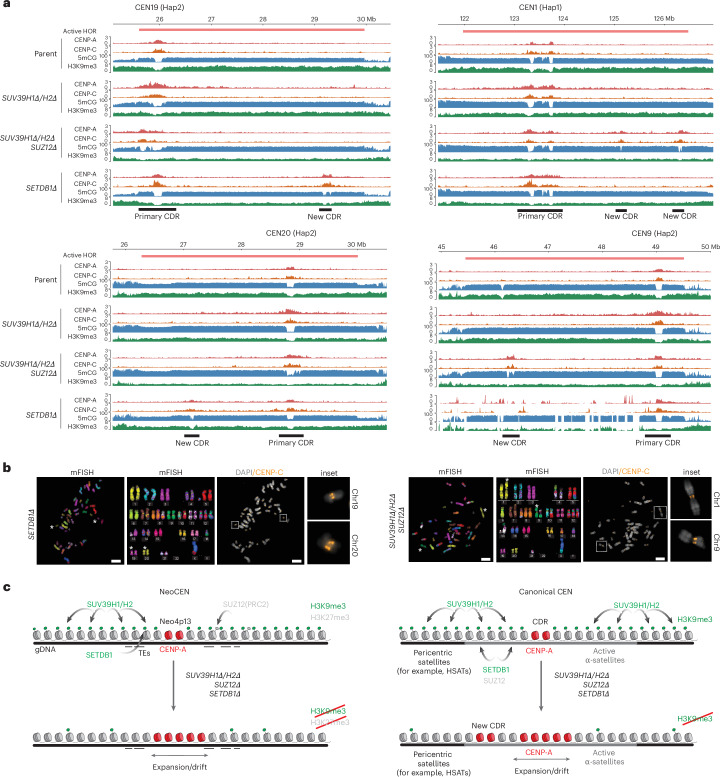
Nucleation of new CENP-A/CENP-C peaks and CDRs, forming functional dicentrics. **a**, Representative genome tracks for the entire alpha-satellites of CEN1 (Hap1), CEN9 (Hap2), CEN20 (Hap2) and CEN19 (Hap2), showing CENP-A (red), CENP-C (orange), 5mCG (blue) and H3K9me3 (green) for parental, *SUV39H1Δ*/*H2Δ*, *SUV39H1Δ/H2Δ/SUZ12Δ* and *SETDBΔ* cell lines. The primary and newly formed centromeres are highlighted in each case, corresponding with new CENP-A and CENP-C seeding and 5mCG and H3K9me3 dips (representative of *n* = 2 independent validations). **b**, Representative immunofluorescence and mFISH images of metaphase spreads, showing karyotype and dicentric chromosomes for *SETDB1Δ* and *SUV39H1Δ/H2Δ/SUZ12Δ* cells as per **a**, identified by CENP-C (orange) and chromosome-specific mFISH paint. Scale bar, 10 μm (*n* = 2 independent experiments, with a minimum of 20 nuclei per replicate). **c**, Model summarizing the differential role of H3K9me3 methyltransferases at neocentromeres and canonical centromeres and their role in defining CENP-A domain size, position and number. Left, loss of SUV39H1/H2-mediated heterochromatin (H3K9me3) and compensatory H3K27me3 (PRC2) at Neo4p13 results in expansion and drift of the neocentromere. SETDB1 contributes to localized H3K9me3, possibly at TEs. Right, loss of H3K9me3 at canonical alpha-satellite centromeres leads to centromere expansion, drift and nucleation of new centromeres at distal sites on the alpha-satellite, creating new CDRs. SUV39H1/H2 broadly contributes to H3K9me3 deposition, while SETDB1 and SUZ12 action is largely restricted to HORs.

In addition to the expansion of existing CENP-A boundaries, we observed many cases where the loss of heterochromatin resulted in entirely new CENP-A peaks. These formed distal to the primary CENP-A domain but within the same alpha-satellite HORs and correlated with the loss of 5mCG and H3K9me3, thus forming characteristically similar chromatin features to the primary active centromere or CDR. For example, CEN1 and CEN9 of *SUV39H1Δ/H2Δ/SUZ12Δ* both contained new CDRs ~3 Mb away from the original primary CDR (Fig. 7a). *SETDB1Δ* cells similarly showed specific examples of new CDRs at CEN19 and CEN20 (Fig. 7a). Furthermore, the presence of the key CCAN component CENP-C indicates that these ectopic CENP-A domains are likely functionally active as centromeres (Fig. 7a). Thorough analysis using CDR-Finder^63^ followed by manual curation (Methods) identified all new CENP-A-containing CDRs that formed distally on the same alpha-satellite HOR in *SUV39H1Δ/H2Δ/SUZ12Δ* and *SETDB1Δ* mutants (Supplementary Table 1) and two new CDRs in *DNMT3BΔ*. A new CENP-A peak was also present on CEN21 Hap1 of parental cells following 100 days of culture, suggesting that new CENP-A domain seeding could be a general phenomenon that occurs at low frequency even in wild-type cells over time. Noticeably, some HOR regions appear to be preferred sites for new CENP-A domain formation across our cell lines (for example, Chr9, Chr20 and Chr21). No new CDRs appeared in *SUV39H1Δ/H2Δ* cells.

**Extended Data Fig. 9:**
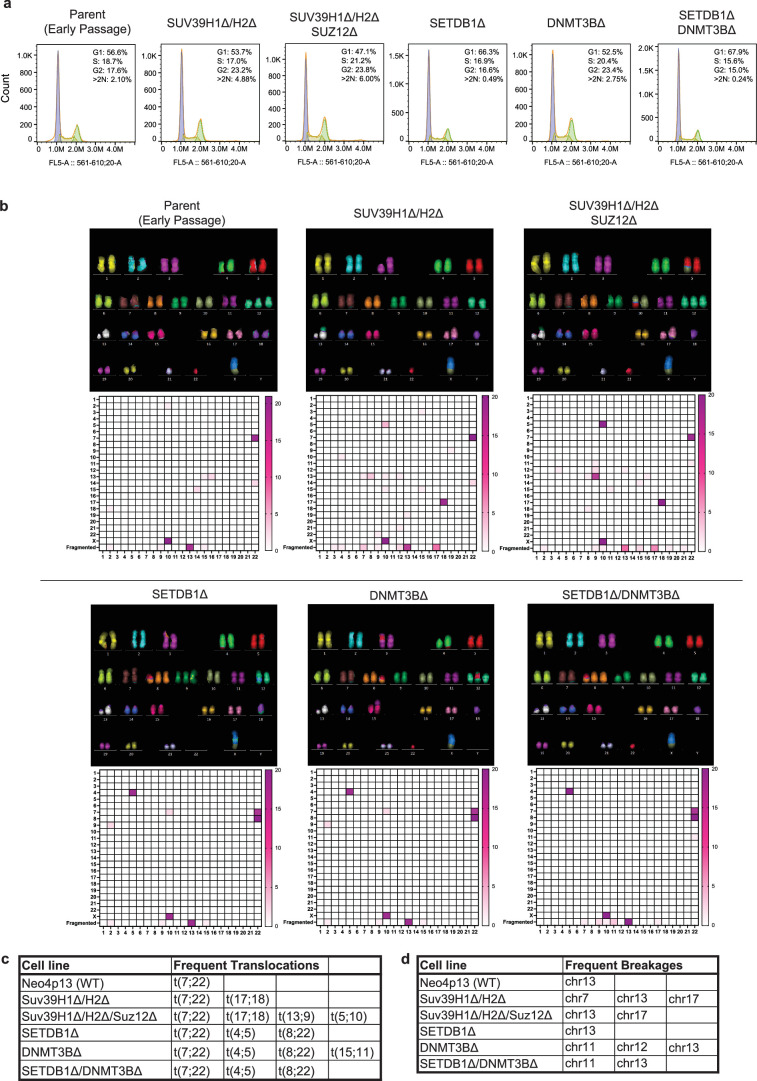
Cell cycle profiles, ploidy and karyotypes of cell lines. **a**, Measure of cell cycle distribution and ploidy by Propidium Iodide DNA-staining and flow cytometry for each cell line used in this study. **b**, Representative mFISH karyotypes for each cell line used in this study, alongside heatmap (below) indicating the frequency of translocations and chromosome breakages for each cell line based on 20 mFISH images per cell line. **c**, Table listing most frequent translocations identified in each cell line. **d**, Table listing most frequent chromosome breakages identified in each cell line. Source data

We found all mutant lines to generally maintain a diploid karyotype as measured by DNA content (Extended Data Fig. 9a), although we observed a small polyploid population in the *SUV39H1Δ/H2Δ* double-mutant and *SUV39H1Δ/H2Δ/SUZ12Δ* triple-mutant cells (Extended Data Fig. 9a). We did not observe new CENP-A domains and CDRs in the *SUV39H1Δ/H2Δ*, indicating that polyploidy of these cells is not directly linked to ectopic CENP-A domains but rather to other defects, induced by heterochromatin loss. Furthermore, using spectral karyotyping, we identified translocations and aneuploidies of specific chromosomes across each cell line (Extended Data Fig. 9b–d). However, we did not find that the translocations resulted from centromeric breakages and appeared to occur independently of new CENP-A peaks. We observed some instances of HOR destabilization not observed in parental cells (for example, CEN9 Hap2 in *SETDB1Δ*; Fig. 7a). However, the majority of HORs appeared stable and translocation events appeared not to be directly linked to centromere defects.

Lastly, to determine whether the ectopic CENP-A peaks form distinct centromere domains, we directly visualized these new centromeres on mitotic chromosomes. Using a combination of multicolor FISH (mFISH) and immunofluorescence for CENP-C, we could resolve dicentric chromosomes in metaphase spreads for chromosomes 1 and 9 of *SUV39H1Δ/H2Δ/SUZ12Δ* mutants and chromosomes 19 and 20 in *SETDB1Δ* cells. Here, CENP-C appeared as distinct double loci at these centromeres (Fig. 7b, orange), corresponding to the new CENP-A and CENP-C DiMeLo-seq peaks (Fig. 7a), indicating that these loci form functional dicentrics with two spatially adjacent CENP-A domains within the same alpha-satellite array.

## Discussion

### Heterochromatin forms a centromere boundary that controls centromere position and size

In this study, we discovered that the human neocentromere Neo4p13 (ref. ^18^) is enriched in SUV39H1-dependent and SUV39H2-dependent H3K9me3 but is formed in generally low levels of DNA methylation and lacks alpha-satellites, indicating that the latter are not the primary contributors to centromere positioning, at least at this neocentromere. We found that both H3K9me3 and redundant H3K27me3 within this domain form effective boundaries, preventing CENP-A domain drift and expansion (Fig. 7c).

We hypothesize that these heterochromatin marks are maintained through self-templated propagation, forming a competitive, incompatible barrier for CENP-A deposition. In this context, it is interesting to note that treatment with an HDAC inhibitor and resulting increase in histone acetylation were also shown to cause CENP-A domain movements at a human neocentromere, an early indicator that changes to heterochromatin affect centromere boundaries^64^. We found that, while loss of SETDB1 had little impact on H3K9me3, it results in movement of the CENP-A domain, indicating that a subtle impact on H3K9me3 levels (particularly at TEs) may have notable consequences for the centromere boundaries (Fig. 7c). Alternatively, SETDB1 may impact other aspects of chromatin, such as H3K9me2, as recently identified at the centromere^65^, or possibly a nonenzymatic role, which requires further investigation.

As at the 4p13 neocentromere, we found H3K9me3 chromatin to be depleted within the CENP-A domains at satellites, correlating with a dip in CpG methylation. Perturbation of these H3K9me3 boundaries resulted in substantial movement and expansion of CENP-A domains at all centromeres. Importantly, while loss of H3K9me resulted in some erosion of CpG methylation at the CDR boundary, the DNA methylation dips were largely maintained. Recent work showed that strong and rapid depletion of DNA methylation within centromeric HORs results in an increase in CENP-A assembly within the CDR and alterations to CENP-A boundary definition^60^. Our work now demonstrates that DNA methylation may not act as the sole boundary to maintain the CENP-A domain and heterochromatin has a critical role in restricting the functional CENP-A domain, particularly at the long timescales we analyzed.

### Differential regulation of centromeric H3K9me3

Centromeric and pericentromeric repeats are largely repressed by H3K9me3-associated chromatin^37,54,55^. How H3K9me3 is regulated within the centromere has remained unclear. Our data support a general role for SUV39 methyltransferases at both pericentric HSat sequences and centromeric alpha-satellites. A role for SETDB1 was also recently observed at centromeres in K562 leukemia cells^65^. We discovered that SETDB1 acts as a methyltransferase primarily at active alpha-satellite HORs (Fig. 7c). SETDB1 is known to drive heterochromatinization and silence TEs^47–49^. While the reason for the HOR-specific effect is unknown, active HORs, like TEs, are highly methylated compared to adjacent inactive arrays^3,28^. Possibly, the high degree of methylation contributes to SETDB1 recruitment. While pure alpha-satellites enriched in CENP-A are usually devoid of TEs^3^, they may nevertheless share similar features. For instance, members of the diverse family of zinc finger DNA-binding factors that normally recruit SETDB1 complexes to TEs were recently implicated in heterochromatin initiation and directly bind pericentric DNA in a sequence-specific manner^66^. Understanding whether cryptic sequences within active HORs contribute toward SETDB1 targeting is an important future goal.

We also identified an important role for the PRC2 component SUZ12 in the regulation of centromeric satellites, independent of H3K27me3, the product of the canonical SUZ12-containing PRC2 complex (Fig. 7c). Indeed, it was previously shown that SUZ12 regulates H3K9 methylation and HP1α distribution in flies^67^. Furthermore, proteomic analyses found PRC2 components enriched on H3K9me3 chromatin, indicating extensive overlap in H3K9me3 and H3K27me3 binding profiles^68,69^. Both EZH2 methyltransferase and SUZ12 appear to be required for HP1 protein stability, and HP1 binding to H3K9me3 peptides is greatly increased in the presence of H3K27me3 (ref. ^70^). On the basis of this functional overlap, loss of SUZ12 may destabilize and erode H3K9me3-marked heterochromatin, consistent with our observations. While we cannot exclude the secondary effects of long-term gene deletions in this study, these observations suggest a possible direct functional interaction among SETDB1, SUV39 and SUZ12 at centromeric repeats, which requires further careful biochemical and genetic dissection.

### Heterochromatin limits the number of CENP-A/CDR domains on alpha-satellite HORs

Prior evidence suggests that centromere nucleation is a concentration-dependent mechanism^5,6^, requiring a critical concentration of CENPs to trigger an active centromere state. Indeed, CENP-A is loaded onto chromatin at low concentrations outside the centromere^40,71,72^ but is removed through DNA replication except at the centromere^71^. A key function of repressive chromatin surrounding the active centromeric locus may be to maintain a high local concentration of CENP-A. Our discovery of new CENP-A domains distal to the primary CDR, which appear to form functional centromeres in heterochromatin-deficient mutants, is consistent with a role for heterochromatin and DNA methylation^60^, not only in acting as a boundary at existing CENP-A domains but also in suppressing aberrant centromere formation at regions outside of the primary active centromere locus. As centromeric transcription appears to be implicated in CENP-A function^73^ and is indeed activated upon centromere repositioning^74^, one possible role of H3K9me3 and DNA methylation may be to constrain centromeric transcription and associated chromatin remodeling specifically within CDRs, where CENP-A assembles.

The new CENP-A domains we observe in heterochromatin mutants occur within the HOR and likely act as functional dicentrics that are mitotically stable and generally permissive to chromosome segregation, consistent with previous work^75^. Nevertheless, multiple CENP-A domains within the HOR may affect mitotic fidelity, particularly inducing merotelically because of a loss of a tight bioriented centromere architecture^76^. Moreover, the recent description of the bipartite organization of the centromere and kinetochore may be impacted by ectopic CENP-A domains, even if proximal to the canonical site, which may impact chromosome segregation fidelity^77^.

In sum, we identified heterochromatin and its associated H3K9 methyltransferases as an important contributor to maintaining CENP-A boundaries. How these boundaries are set at the remarkably constant size of about 100 kb, independent of underlying sequence context, remains an open question. Possibly, the assembly of the multiprotein centromere complex and the functional kinetochore tie into control mechanisms dynamically dictating the number of CENP-A nucleosomes, as we recently discussed^78^. Understanding the dynamic feedback involving CENP-A chromatin, its boundaries and the centromere–kinetochore complex is an important future goal.

## Methods

### Cell line and culture conditions

All human cell lines were grown at 37 °C, 5% CO_2_. RPE1 cell lines were grown in DMEM/F-12 medium (Gibco) supplemented with 10% FBS (Gibco), 1× nonessential amino acids (Gibco), 2 mM glutamine (Gibco), 100 U per ml penicillin (Gibco) and 100 µg ml^−1^ streptomycin (Gibco). HEK293 cells were grown in DMEM (Gibco) supplemented with 10% FBS (Gibco).

### Cell line generation and lentiviral transduction

The RPE-Neo4p13 cell line was generated as previously described^18^. For *SUV39H1/H2*, *SETDB1* and *DNMT3B* deletion in the RPE-Neo4p13 background, guide RNAs (gRNAs) were designed (Supplementary Table 2; synthesized by Merk Life Sciences) and cloned into LentiCRISPRv2-Blast (Addgene, 83480) according to the standard lentiCRISPRv2 cloning protocol^79^. Lentivirus was produced in HEK293T cells in a T25 flask using a plasmid mixture (0.62 µg of pMD2 (Addgene, 12259), 1.87 µg of psPAX2 (Addgene, 12260) and 2.5 µg of pLentiCRSPRv2), transfected with Lipofectamine LTX. The virus supernatant was then harvested, filtered through a 0.45-µm filter and either frozen at −80 °C or used immediately. For transduction, RPE-Neo4p13 cells were seeded at 5 × 10^4^ cells per well in a six-well plate the day before infection. Filtered viral supernatant was mixed with antibiotic-free medium, supplemented with 3.2 µl of 10 mg ml^−1^ polybrene and added to the cells for 48 h. After infection, cells were washed four times with PBS, replaced with regular medium and, if required, treated with the 5 µg ml^−1^ blasticidin for 5 days. Cells were then clonally sorted into 96-well plates, grown out and screened by immunoblot for positive clonal knockouts for each target.

For generation of the *SUV39H1Δ/H2Δ/SUZ12Δ* cell line, *SUZ12* gRNAs (Supplementary Table 2) were designed and cloned into pSpCas9(BB)-2A-GFP (PX458) plasmid using the standard Zhang lab gRNA insertion protocol^80^. Clonal *SUV39H1Δ/H2Δ* cells were transiently reverse cotransfected with PX458 using the Mirus TransIT-LT1 protocol at a 2:1 ratio of reagent to DNA in a six-well plate at a density of 6 × 10^4^ cells per well. After 48 h, cells were sorted for high GFP expression into a polyclonal population and allowed to recover for 1 week. When cells were proliferating, cells were clonally sorted (GFP-negative) into 96-well plates and allowed to expand, followed by screening by immunoblot for clones in which both SUZ12 and resulting H3K27me3 were lost.

### Fluorescence-activated cell sorting (FACS), flow cytometry and cell-cycle analysis

For FACS and cytometry, cells were harvested by centrifuging at 500*g* for 5 min and resuspended in ice-cold sorting medium, which consisted of 1% FBS in PBS, supplemented with 0.25 mg ml^−1^ Fungizone (Thermo Fisher Scientific) and 0.25 μg ml^−1^ amphotericin B and 10 μg ml^−1^ gentamicin (Gibco). The cell suspension was filtered through 5-ml polystyrene round-bottom tubes equipped with cell-strainer caps (Falcon) before sorting and analysis using the FACSAria III or FACSAria Fusion cell sorter (BD Biosciences). Sorted cells were collected into 96-well plates containing conditional medium, prepared as a 1:1 mix of fresh complete medium and 0.45-μm-filtered medium from proliferating cell cultures, supplemented with 20% FBS, 0.25 mg ml^−1^ Fungizone (Thermo Fisher Scientific), 0.25 μg ml^−1^ amphotericin B and 10 μg ml^−1^ gentamicin (Gibco).

For cell-cycle analysis, cells were harvested and washed with PBS. Fixation was performed by adding 1 ml of cold 70% ethanol dropwise to the cell pellet while vortexing to ensure thorough fixation and minimize clumping. Cells were fixed for at least 30 min on ice. Fixed cells were washed twice with PBS. To selectively stain DNA, cells were treated with 50 µl of RNase A, followed by the addition of 400 µl of propidium iodide solution (50 µg ml^−1^) per million cells directly to the RNase-A-treated pellet. The samples were mixed thoroughly and incubated at room temperature for 10 min. The stained samples were analyzed by flow cytometry and data were collected from at least 20,000 single cells per condition. Data were analyzed using FlowJo version 10.8 (BD Life Sciences).

### Immunoblotting

Cell pellets were resuspended in RIPA buffer, incubated for 20 min on ice and then centrifuged at 15,000*g* for 20 min at 4 °C. The supernatant was transferred to a fresh Eppendorf tube; protein concentration was measured with a Bradford assay and adjusted to the same concentration. Samples were resuspended to 1× sample buffer (125 mM Tris–HCl pH 6.8, 10% glycerol, 1% SDS, 0.2% (w/v) orange G and 10% β-mercaptoethanol) and boiled at 95 °C for 5 min. Then, 10 µg of protein was loaded per lane in a 4–20% SDS–PAGE gel (BioRad), followed by transfer to a nitrocellulose membrane (BioRad Transblot Turbo). Membranes were blocked in 5% (w/v) milk or BSA in TBS-T (20 mM Tris–HCl pH 7.5, 150 mM NaCl and 0.1% Tween-20) for 1 h and incubated with primary antibodies at 4 °C overnight with gentle rocking. Membranes were washed three times with TBS-T for 10 min each, followed by incubation with secondary antibody diluted in 5% (w/v) milk or BSA in TBS-T for 1 h at room temperature with gentle rocking, followed by washing three times with TBS-T for 10 min each. For fluorescent antibodies, membranes were visualized with an Odyssey Fc gel imager (LI-COR). For horseradish-peroxidase-conjugated secondaries (Extended Data Fig. 3b), enhanced chemiluminescence reagent (BioRad Clarity ECL reagent, 1705061) was used for detection and membranes were visualized on X-ray films (Cytiva, GE28-9068-37), developed on an OPTIMAX 2010 X-ray film processor (PROTAC-Med). Primary and secondary antibodies are listed in Supplementary Table 3.

### CUT&RUN sequencing and library preparation

CENP-A, H3K9me3 and H3K27me3 CUT&RUN were performed natively with 5 × 10^5^ cells using the CUTANA ChIC/CUT&RUN kit (EpiCypher, 14-1048) according to the manufacturer’s protocol (version 3, 4 or 5). The following antibodies and concentrations were used: CENP-A (Enzo, ADI-KAM-CC006, 1:50), H3K9me3 (Abcam, ab8898, 1:50) and H3K27me3 (C36B11, 1:50) (Supplementary Table 3). DNA sequencing libraries were prepared using the NEBNext Ultra II DNA library prep kit for Illumina (New England Biolabs) following the published protocol. For multiplexing, we used NEBNext multiplex oligos for Illumina (index primer sets 1 and 2). Size selection was performed using Ampure XP beads (Beckman Coulter) to isolate mononucleosomal DNA fragments of approximately 150–180 bp (excluding adaptors). Library yield and quality were evaluated using the Qubit HS double-stranded DNA quantification assay kit (Thermo Fisher Scientific) and the TapeStation 4150 System (Agilent). Multiplexed libraries were diluted to final concentrations of 1, 2 or 4 nM and sequenced at 1.8 pM on a NextSeq 500 system (Illumina) with the NextSeq 500/550 high output version 2.5 (75 cycles) kit (Illumina).

For the analysis of CUT&RUN data, raw FASTQ files were downloaded and concatenated per sample using basic Unix commands. Read quality was assessed using Basespace (Illumina) and FastQC software^81^. Reads were then aligned to the RPE1 genome using Bowtie2 (ref. ^82^), with trimming parameters adjusted on the basis of read quality. SAM-to-BAM file conversion, BAM sorting and indexing were carried out using SAMtools (version 1.16)^83^. Duplicate reads were removed using the MarkDuplicates command in Picard (https://broadinstitute.github.io/picard/). The resulting sorted and duplicate-removed BAM files were used to calculate read counts, normalize data (counts per million) and convert to BigWig format using the bamCoverage command in deepTools (version 2)^84^. BigWig files were visualized using Integrative Genomics Viewer and with pyGenomeTracks^85^. Read count matrices were generated using the multiBigWigSummary command in deepTools (version 2) and the output was visualized using matplotlib.

### MBP–nanobody–Hia5–His6 Purification

Mouse and Rabbit MBP–nanobody–Hia5–6×His constructs were a gift from A. Straight (Stanford University)^86^. MBP–nanobody–Hia5 proteins were overexpressed by rhamnose induction in BL21 DE3 cells for 16–20 h at 18 °C before harvesting by centrifugation. Cells were pelleted and resuspended in lysis buffer (50 mM HEPES pH 7.5, 300 mM NaCl, 10% glycerol and 0.5% Triton X-100) with one ETDA-free protease inhibitor tablet per liter of culture. The cells were then lysed by probe sonication (Qsonica Q125; six pulses, 30 s on and 1 min off at 200 W). After a clearance centrifugation at 40,000*g* for 1 h, the supernatant was added to washed Ni-NTA beads and mixed for 1 h at 4 °C. The slurry was added to a gravity flow column and washed with 50 mM HEPES pH 7.5 and 300 mM NaCl followed by 50 mM HEPES pH 7.5, 300 mM NaCl and 20 mM imidazole. The protein was eluted by 50 mM HEPES pH 7.5, 300 mM NaCl and 250 mM imidazole in 30ml and confirmed by SDS–PAGE. The elution was then dialyzed to remove the imidazole at 4 °C overnight in 50 mM Tris–HCl, pH 8.0, 100 mM NaCl and 1 mM DTT. After dialysis, the protein was concentrated, filtered and applied to a Recourse Q anion-exchange column. After confirmation of protein purity by SDS–PAGE, the protein was concentrated to 1 mg ml^−1^, snap-frozen and stored at −80 °C until use.

### DiMelo-seq and high-molecular-weight (HMW) DNA extraction

DiMeLo-seq was performed according to the standard protocol in Maslan et al.^87^ with some minor changes; all buffer compositions followed the published protocol. Briefly, 6 × 10^6^ RPE cells were pelleted and resuspended in 1× PBS. For acute IAA experiments and CENP-C DiMeLo-seq, cells were fixed with 0.1% paraformaldehyde (PFA) by adding 6.2 µl of 16% PFA to 1 ml of suspension, followed by gentle vortexing for 2 min. Fixation was quenched with 1.25 M glycine (60 µl per 1 ml of suspension), followed by centrifugation at 500*g* for 3 min at 4 °C, supernatant removal and resuspension in Dig-Wash buffer (0.02% digitonin). All other samples were performed natively as per the published protocol (antibodies and concentrations in Supplementary Table 3).

HMW genomic DNA was extracted using the NEB Monarch HMW DNA extraction kit for tissue, resuspending in a final volume of 750 µl of EEB buffer (ONT). This 750-µl volume was then taken forward to the tagmentation step as per the standard SQK-ULK114 protocol. CENP-A and H3K9me3 DiMeLo-seq DNA was sequenced on a PromethION 2 Solo device (ONT) with R10.4.1 flow cells with adaptive sampling for all centromeres and all of Chr4 to depths of 25 Gb (CENP-A and Mouse IgG1κ) and 20 Gb (H3K9me3, H3K27me3, CENP-C and rabbit IgG).

### Data processing of DiMelo-seq

Pod5 files were basecalled using dorado version 0.7.3 using the dorado basecaller sup@v4.3.0 for 6mA and 5mCG/5hmCG (https://github.com/nanoporetech/dorado). Basecalled BAM files were then aligned to the RPE1 genome^39^ using dorado. Aligned BAM files were then sorted using SAMtools (version 1.16.1), filtered with SAMtools view -F 2308 to remove unmapped alignments (0 × 4), nonprimary alignments (0 × 100) and supplementary alignments (0 × 800), following by indexing. Indexed BAM files were then processed through a refModMatch script (https://github.com/altemoselab/miscTools/blob/main/refModMatch.py) to ensure we only kept bases in the MM tag if they were a match to the reference genome.

Next, 6mA and 5mCG modification filtering was performed using modkit (https://github.com/nanoporetech/modkit) using modkit pileup with the following thresholds: --filter-threshold A:0.8 --mod-threshold a:0.98 --filter-threshold C:0.8 --mod-threshold m:0.8. The 6mA thresholds were tuned for specificity and signal-to-noise ratio using endogenous CENP-A auxin depletion (Extended Data Fig. 3) where endogenous CENP-A was fully depleted. BigWig files for visualization were generated from columns 1, 2, 3 and 11 (fraction modified) of the output bedmethyl file from modkit. To visualize single molecules, we used fibertools-rs (version 0.5.4; https://github.com/fiberseq/fibertools-rs)^88^ and used the ft extract tool to extract --m6A and --cpg motifs into a BED file with --ml 250 and --ml 204 thresholds, respectively, for each modification (in line with modkit filtering above). BigWig files and single-molecule tracks were visualized with pyGenomeTracks^85^.

### Centromere annotation and identification of HORs and CDRs

Alpha-satellite annotations were previously identified using HumAS-HMMER For AnVIL (https://github.com/fedorrik/HumAS-HMMER_for_AnVIL)^39^. The resulting annotations were compared with CHM13 CenSat annotations^3^ to identify active, inactive and divergent HORs, as well as monomeric satellites, in the RPE1 genome. For analysis of active HORs in Figs. 4 and 5, active HOR annotations were lifted to include only the CDR/CENP-A-containing active HOR. Cases such as Cen3 (Hap1 and Hap2), Cen4 (Hap1) and Cen13 (Hap2), which contain multiple split active HORs, were contained to just the CENP-A-containing HOR. These CENP-A-containing active HORs were normalized to the same length, as described below, and plotted as active HOR ± 1 Mb, with flanking regions broadly defined as ‘pericentromere’.

### Normalization of active HOR and CDR length

To standardize regions of varying lengths for heat map analysis, each region was normalized to a fixed number of bins. Regions were extended symmetrically with upstream and downstream flanking areas. For active HORs, regions were normalized to 1,000 bins, with flanking pericentromeric regions (±1 Mb) divided into 500 bins each. CDRs were scaled to 200 bins, while each flank (±125 kb) was divided into 125 bins. Mean signal intensities for each bin were extracted from BigWig files using the pyBigWig.stats() function, with missing values replaced by NaN. The normalized signal profiles for all regions were combined into matrices for visualization, where each row represents a single active HOR or CDR, sorted numerically (for example, chr1_hap1, chr1_hap2, chr2_hap1, etc.).

### Centromere length analysis, new CDR detection and 6mA density analysis

CDRs were initially identified with CDR-Finder (https://github.com/EichlerLab/CDR-Finder)^63^ using the following parameters: height_perc_valley_threshold, 0.39; prom_perc_valley_threshold, 0.45. All other parameters were as per default test parameters. After initial CDR detection, each CDR BED file was manually inspected for accuracy and compared to CENP-A DiMelo-seq data. Any non-CENP-A-containing CDRs were manually removed. CDRs for Chr7 Hap1 and Chr10 Hap2 were manually annotated for accuracy. To ensure comparison of only primary CDRs, all nonprimary and newly formed CDRs were removed. The total length of each CDR was then summed and directly compared on a per-centromere basis to the corresponding parent centromere. To detect newly formed CDRs, the CDR-Finder^63^ output was manually inspected as above for accuracy, without removing nonprimary CDRs. All CDRs within 500 kb were merged and considered as one single centromere locus. Any CENP-A-containing CDRs not present in the parent CDR database were considered newly formed (that is, any new centromere peak more than 500 kb from the primary CDR site). The following chromosomes were not considered in all CDR analyses: Cen4 Hap2 (neocentromere-containing haplotype), CENX Hap2 (not present in genome), Cen18 Hap1 and Cen18 Hap2 (poorly defined CDRs).

CENP-A and CENP-C peaks were identified using a seeded expansion approach from preannotated CDRs (above). For each BigWig file containing 6mA/A-modified fraction data, the mean signal within each CDR was calculated and a threshold was set at 1% of this mean. A genomic window extending 30 kb beyond the CDR boundaries was loaded, with the signal smoothed using a 2-kb rolling average to mitigate noise. Starting from the CDR midpoint, boundaries were expanded left and right until the smoothed CENP-A/CENP-C signal fell below the threshold. Resulting peak annotations for each centromere were sorted and output as BED files.

To quantify 6mA density in detected CENP-A peaks, regions enriched for CENP-A relative to IgG controls, DiMeLo-seq reads in BED12 format from the fibertools extract were processed for each sample (parent, *SUV39H1/H2Δ*, *SUV39H1/H2Δ/SUZ12Δ* and *SETDB1Δ*). The BED12 file output from the fibertools extract provided the read’s chromosome, start and end positions (columns 1–3), read name (column 4), block count (column 10, representing the number of 6mA sites), block sizes (column 11, typically 1 for point modifications) and relative block starts (column 12, indicating 6mA positions relative to the read start). Reads overlapping sample-specific CDR intervals by at least 10 kb (after excluding chromosomes chr4_2, chr18, chrX_2, chr13_2 and chr21_2) were identified and 6mA counts within these overlaps were tallied per molecule by checking whether each block’s absolute position fell within the CDR boundaries. Density was computed as the number of 6mA sites per 10 kb of overlapping CDR sequence. Enrichment was calculated by dividing CENP-A densities by corresponding IgG control densities and plotted with matplotlib and seaborn.

### Mitotic spreads

Cells were treated with KaryoMAX Colcemid (100 ng ml^−1^, Gibco) for 3 h and mitotic cells were collected by mitotic shakeoff. To prepare cells for spreading, a 75 mM KCl solution was prewarmed in a 37 °C water bath and a fresh Carnoy fixative solution (methanol and acetic acid, 3:1) was prepared and placed on ice. The cell suspension was centrifuged at 500*g* for 5 min and the supernatant was discarded, leaving a small residual volume in which the pellet was gently resuspended. For hypotonic treatment, 1 ml of 75 mM KCl (prewarmed to 37 °C) was slowly added to the cell suspension, followed by an additional 4 ml of the same solution. The tubes were incubated in a 37 °C water bath for 15 min, with gentle shaking once or twice during incubation to homogenize the solution. After incubation, the cells were centrifuged at 500*g* for 5 min at 4 °C. The supernatant was removed, leaving approximately 500 μl with the cell pellet and the tubes were immediately placed on ice.

To fix the cells, the pellet was gently resuspended in the residual supernatant. While the tube was vortexed at the lowest speed, 1,000 μl of ice-cold fixative was added dropwise, followed by an additional 10 ml of fixative. The suspension was centrifuged at 230*g* for 10 min at 4 °C and the cell pellet was resuspended in 10 ml of fresh fixative. The prepared cells were stored at −20 °C until ready for chromosomal spreading.

For dropping onto slides, Epredia Superfrost Plus slides were cleaned and kept in a coplin jar with methanol and kept at 4 °C until required. When ready for dropping, a cleaned slide was removed and placed tilted on an Eppendorf tube rack. Then, 100 μl of fixed cells were dropped from a ~30-cm height onto the slide and allowed to flow down the glass. Excess fixative was removed on a paper towel and the slide was allowed to dry fully for ~5 min.

### Immunofluorescence and mFISH karyotyping

Immunofluorescence on cells fixed in methanol and acetic acid was performed as described in Beh et al.^89^, with some modifications. The primary antibody used to identify the dicentric chromosome was a guinea pig anti-CENP-C (1:1,000, MBL, PD030) and the secondary antibody was used at 1:500 (anti-guinea pig Alexa Fluor 488, Jackson ImmunoResearch Laboratories). The Metafer imaging platform (MetaSystems, Metafer4 version 3.13.5) and Isis software (version 5.8.14) were used for automated acquisition of the chromosome spreads. For mFISH following immunocytochemistry, slides were washed in PBS for 3 min as described in Beh et al.^89^ and mFISH was performed with a 24 XCyte probe (MetaSystems) following the manufacturer’s instructions. The Metafer imaging platform (MetaSystems, Metafer4 version 3.13.5) and Isis software (version 5.8.14) were used for the second acquisition of chromosome spreads and mFISH image analysis.

## Resource availability

### Materials availability

Requests for cell lines, plasmids or other resources generated in this study should be directed to L.E.T.J. (lars.jansen@bioch.ox.ac.uk).

### Reporting summary

Further information on research design is available in the Nature Portfolio Reporting Summary linked to this article.

## Online content

Any methods, additional references, Nature Portfolio reporting summaries, source data, extended data, supplementary information, acknowledgements, peer review information; details of author contributions and competing interests; and statements of data and code availability are available at 10.1038/s41594-025-01706-2.

## Supplementary information

Supplementary Information Tables with coordinates of all new CENP-A-containing CDRs, gRNA primers and antibodies used.

Reporting Summary

## Source data

Source Data Fig. 1 MultiBigWig summary output and data for bar plots.

Source Data Fig. 3 MultiBigWig summary output and data for bar plots.

Source Data Fig. 3 Uncropped blots.

Source Data Fig. 6 Nanopore sequencing source data, centromere length source data and chromosome enrichment summaries.

Source Data Extended Data Fig. 2 Uncropped blots.

Source Data Extended Data Fig. 7 Uncropped blots.

Source Data Extended Data Fig. 9 mFISH source data.

## Data Availability

All sequencing data were deposited to the European Nucleotide Archive under accession number PRJEB85119. Processed data files and annotations are available through BioStudies under accession number S-BSST2137. This version supersedes a previous submission (S-BSST1935) associated with the *bioRxiv* version of this paper. Source data are provided with this paper.

## Code availability

The scripts for the bioinformatic analyses with their parameters were deposited to a public GitHub repository (https://github.com/bencarty2/Cartyetal_heterochromatin-boundaries).
