# Author Correction: Heterochromatin boundaries maintain centromere position, size and number

**DOI:** 10.1038/s41594-026-01748-0

**Published:** 2026-01-21

**Authors:** Ben L. Carty, Danilo Dubocanin, Marina Murillo-Pineda, Marie Dumont, Emilia Volpe, Pawel Mikulski, Julia Humes, Oliver Whittingham, Daniele Fachinetti, Simona Giunta, Nicolas Altemose, Lars E. T. Jansen

**Affiliations:** 1 Dept of Biochemistry, University of Oxford, Oxford, UK; 2 Department of Genetics, Stanford University, Palo Alto, CA USA; 3 UMR144 and UMR3664, CNRS, Institut Curie, PSL Research University, Sorbonne Université, Paris, France; 4 Laboratory of Genome Evolution, Department of Biology and Biotechnologies ‘Charles Darwin’, University of Rome ‘La Sapienza’, Rome, Italy; 5 Present Address: Infectious Disease and Microbiology Unit. Biomedical Innovation and Research Institute of Cádiz (INiBICA), University Hospital of Jerez, University of Cádiz, Cádiz, Spain; 6 Present Address: Nencki Institute of Experimental Biology, Polish Academy of Sciences, Warsaw, Poland

**Keywords:** DNA methylation, Epigenomics, Chromosome segregation, Histone post-translational modifications, Centromeres

Correction to: *Nature Structural & Molecular Biology* 10.1038/s41594-025-01706-2, published online 25 November 2025.

In the version of this article initially published, in Fig. 1f, the color keys for “Left (–90 kb)” and “Right (+90 kb),” currently appearing as dark grey and light grey, respectively, were incorrectly swapped. In Fig. 3d, the data presented were inadvertent duplicates of Fig. 3j. Figures 1f and 3d have now been amended in the HTML and PDF versions of the article.

