# Supplementary material for: Heterochromatin boundaries maintain centromere position, size and number
Source: Nat Struct Mol Biol. 2025 Nov 25;33(2):220–34. doi: 10.1038/s41594-025-01706-2 (PMC7618434; doi:10.1038/s41594-025-01706-2)
Supplement: Supplementary file 1 — Tables with coordinates of all new CENP-A-containing CDRs, gRNA primers and antibodies used. [file 41594_2025_1706_MOESM1_ESM.pdf]

nature structural & molecular biology

Supplementary information https://doi.org/10.1038/s41594-025-01706-2

Heterochromatin boundaries maintain centromere position, size and number

In the format provided by the authors and unedited

Supplemental Tables

Cell Line	Chromosome	Start	End
Parent 100d Clone 1.2	Chr21 (Hap1)	4830300	4895300
SUV39H1Δ/H2Δ/SUZ12Δ	Chr1 (Hap1)	125004534	125149534
SUV39H1Δ/H2Δ/SUZ12Δ	Chr1 (Hap1)	126269534	126354534
SUV39H1Δ/H2Δ/SUZ12Δ	Chr9 (Hap2)	46215681	46365681
SUV39H1Δ/H2Δ/SUZ12Δ	Chr20 (Hap1)	27068226	27113226
SUV39H1Δ/H2Δ/SUZ12Δ	Chr21 (Hap1)	4650300	4860300
SUV39H1Δ/H2Δ/SUZ12Δ 100d Clone 1.2	Chr1 (Hap1)	125109534	125149534
SUV39H1Δ/H2Δ/SUZ12Δ 100d Clone 1.2	Chr1 (Hap1)	126269534	126419534
SUV39H1Δ/H2Δ/SUZ12Δ 100d Clone 1.2	Chr3 (Hap2)	96531917	96561917
SUV39H1Δ/H2Δ/SUZ12Δ 100d Clone 1.2	Chr9 (Hap2)	46215681	46360681
SUV39H1Δ/H2Δ/SUZ12Δ 100d Clone 1.2	Chr20 (Hap1)	27063226	27128226
SUV39H1Δ/H2Δ/SUZ12Δ 100d Clone 1.2	Chr21 (Hap1)	4645300	4865300
SETDB1Δ	Chr7 (Hap1)	65533216	65578216
SETDB1Δ	Chr9 (Hap1)	45447340	45487340
SETDB1Δ	Chr19 (Hap2)	29111633	29296633
SETDB1Δ	Chr20 (Hap2)	27113480	27193480
SETDB1Δ	Chr21 (Hap1)	4825300	4895300
DNMT3BΔ	Chr9 (Hap2)	46100681	46170681
DNMT3BΔ	Chr21 (Hap1)	4830300	4895300
SETDB1Δ/ DNMT3BΔ	Chr9 (Hap2)	49140681	49325681
SETDB1Δ/ DNMT3BΔ	Chr19 (Hap2)	29116633	29321633
SETDB1Δ/ DNMT3BΔ	Chr20 (Hap2)	27088480	27188480
SETDB1Δ/ DNMT3BΔ	Chr21 (Hap1)	4835300	4900300

Table 1: Summary of the coordinates of all new CENP-A containing CDRs within HOR regions as defined by CENP-A DiMeLo-seq.

Vector	Target	Forward	Reverse
pLentiCRISPRv2-Blast	*SUV39H1*	CACCGGATCTTCTTGTAATCGCAC	AAACGTGCGATTAACAAGAAGATCC
pLentiCRISPRv2-Blast	*SUV39H2*	CACCGTTTCGAACTAGCAATGGACG	AAACCGTCCATTGCTAGTTCGAAAC
pLentiCRISPRv2-Blast	*SETDB1*	CACCGCCTTCCTGGGTGCATTGGTT	AAACAACCAATGCACCCAGGAAGGC
pLentiCRISPRv2-Blast	*DNMT3B*	CACCGAGAGTCGCGAGCTTGATCTT	AAACAAGATCAAGCTCGCGACTCTC
PX458	*SUZ12* gRNA 1<br>*SUZ12* gRNA 2	CACCGCAGTTCACTCTTCGTTGGAC<br>CACCGCAGTTCACTCTTCGTTGGAC	AAACAGGAGCCGTAGATTTATCATC<br>AAACGTCCAACGAAGAGTGAACTGC

Table 2: Table of gRNA Primers used for CRISPR knockouts. Bsmbi and Bbsi compatible overhangs are underlined.

Target	Antibody	Working dilution	Assay
SUV39H1	Cell Signalling (D11B6)	1:1000	Immunoblot
SUV39H2	Abcam (ab190870)	1:1000	Immunoblot
SUZ12	Cell Signalling (D39F6)	1:1000	Immunoblot
SETDB1	Cell Signalling (C1C12)	1:1000	Immunoblot
CENP-A	Cell Signalling (2186S)	1:1000	Immunoblot
Histone H4	Merck Millipore (05-858)	1:1000	Immunoblot
DNMT3B	Cell Signalling (E2Q3Z)	1:800	Immunoblot
α-tubulin	Sigma (T9026)	1:5000	Immunoblot
H3K9me3	Abcam (ab8898)	1:1000	Immunoblot
H3K27me3	Cell Signalling (C36B11)	1:1000	Immunoblot
Rabbit IgG	IRDye® 800CW (926-32211)	1:5000	Immunoblot
Mouse IgG	Rockland DyLight™ 680 (610-744-124)	1:5000	Immunoblot
Rabbit IgG	Jackson ImmunoResearch HRP (111-035-008)	1:5000	Immunoblot
CENP-A	Enzo (ADI-KAM-CC006)	1:50 @ 1mg/mL	DiMeLo-Seq
H3K9me3	Abcam (ab8898)	1:50 @ 1mg/mL	DiMeLo-Seq
H3K27me3	Cell Signalling (C36B11)	1:50 @ 0.1mg/mL	DiMeLo-Seq
CENP-C	Gift from Don Cleveland, described in Mitra *et al*, 2020 *Nat Comms*; (Human CENP-C aa1-198, Mouse IgG1k)	1:50 @ 1mg/mL	DiMeLo-Seq
Mouse IgG1	Cell Signalling (G3A1)	1:125 @ 2.5mg/mL	DiMeLo-Seq
Rabbit IgG	Cell Signalling (2729)	1:500 @ 1mg/mL	DiMeLo-Seq
CENP-A	Enzo (ADI-KAM-CC006)	1:50 @ 1mg/mL	CUT&RUN
H3K9me3	Abcam (ab8898)	1:50 @ 1mg/mL	CUT&RUN
H3K27me3	Cell Signalling (C36B11)	1:50 @ 0.1mg/mL	CUT&RUN
Rabbit IgG	EpiCypher (13-0042)	1:50 @ 0.5,g/mL	CUT&RUN

Table 3: Antibodies used for Immunoblotting, DiMeLo-Seq and CUT&RUN.
