# Supplementary material for: Heterochromatin boundaries maintain centromere position, size and number
Source: Nat Struct Mol Biol. 2025 Nov 25;33(2):220–34. doi: 10.1038/s41594-025-01706-2 (PMC7618434; doi:10.1038/s41594-025-01706-2)
Supplement: Supplementary file 2 — Reporting Summary [file 41594_2025_1706_MOESM2_ESM.pdf]

nature portfolio

Corresponding author(s): Lars E.T. Jansen
NSMB-A50683A

Last updated by author(s): Sep 30, 2025

Reporting Summary

Nature Portfolio wishes to improve the reproducibility of the work that we publish. This form provides structure for consistency and transparency in reporting. For further information on Nature Portfolio policies, see our Editorial Policies and the Editorial Policy Checklist.

Statistics

For all statistical analyses, confirm that the following items are present in the figure legend, table legend, main text, or Methods section.

n/a	Confirmed	
☐	☒	The exact sample size (n) for each experimental group/condition, given as a discrete number and unit of measurement
☐	☒	A statement on whether measurements were taken from distinct samples or whether the same sample was measured repeatedly
☒	☐	The statistical test(s) used AND whether they are one- or two-sided<br>*Only common tests should be described solely by name; describe more complex techniques in the Methods section.*
☐	☒	A description of all covariates tested
☐	☒	A description of any assumptions or corrections, such as tests of normality and adjustment for multiple comparisons
☐	☒	A full description of the statistical parameters including central tendency (e.g. means) or other basic estimates (e.g. regression coefficient) AND variation (e.g. standard deviation) or associated estimates of uncertainty (e.g. confidence intervals)
☒	☐	For null hypothesis testing, the test statistic (e.g. F, t, r) with confidence intervals, effect sizes, degrees of freedom and P value noted<br>*Give P values as exact values whenever suitable.*
☒	☐	For Bayesian analysis, information on the choice of priors and Markov chain Monte Carlo settings
☒	☐	For hierarchical and complex designs, identification of the appropriate level for tests and full reporting of outcomes
☒	☐	Estimates of effect sizes (e.g. Cohen's d, Pearson's r), indicating how they were calculated

Our web collection on statistics for biologists contains articles on many of the points above.

Software and code

Policy information about availability of computer code

Data collection	The scripts for the bioinformatic analyses with their parameters were deposited in public repository on GitHub under the link: https://github.com/bencarty2/Cartyetal_heterochromatin-boundaries. Data collection was performed with basespace-cli using scripts provided on aforementioned GitHub page.
Data analysis	The scripts for the bioinformatic analyses with their parameters were deposited in public repository on GitHub under the link: https://github.com/bencarty2/Cartyetal_heterochromatin-boundaries. Data analysis was performed with unix-based tools: FastQC v0.12.1, Bowtie2 v2.5.1, Samtools v1.17, Picard v2.27, Deeptools v3.5.5. Nanopore sequencing analysis was performed with Dorado v7.3, modkit v0.4.0, fibertools-rs v0.5.4 and CDR-Finder v1.0.0. Sequencing data was generated and visualised in Jupyter Notebook with pyGenomeTracks, pyBigWig, matplotlib and seaborn python3 packages. FACS data was analyzed using FlowJo™ v10.8 Software (BD Life Sciences).

For manuscripts utilizing custom algorithms or software that are central to the research but not yet described in published literature, software must be made available to editors and reviewers. We strongly encourage code deposition in a community repository (e.g. GitHub). See the Nature Portfolio guidelines for submitting code & software for further information.

Data

Policy information about availability of data

All manuscripts must include a data availability statement. This statement should provide the following information, where applicable:

- Accession codes, unique identifiers, or web links for publicly available datasets
- A description of any restrictions on data availability
- For clinical datasets or third party data, please ensure that the statement adheres to our policy

All data related to this manuscript is publicly available. Sequencing data have been deposited in the European Nucleotide Archive (ENA) under accession number PRJEB85119. Processed data files and annotations are available via BioStudies under accession S-BSST2137, which supersedes a previous submission S-BSST1935. The version of the code used for this study is available at https://github.com/bencarty2/Cartyetal_heterochromatin-boundaries_v1.

Research involving human participants, their data, or biological material

Policy information about studies with human participants or human data. See also policy information about sex, gender (identity/presentation), and sexual orientation and race, ethnicity and racism.

Reporting on sex and gender	n/a
Reporting on race, ethnicity, or other socially relevant groupings	n/a
Population characteristics	n/a
Recruitment	n/a
Ethics oversight	n/a

Note that full information on the approval of the study protocol must also be provided in the manuscript.

Field-specific reporting

Please select the one below that is the best fit for your research. If you are not sure, read the appropriate sections before making your selection.

☒ Life sciences ☐ Behavioural & social sciences ☐ Ecological, evolutionary & environmental sciences

For a reference copy of the document with all sections, see nature.com/documents/nr-reporting-summary-flat.pdf

Life sciences study design

All studies must disclose on these points even when the disclosure is negative.

Sample size	For evolution experiments, we culture 3 independent plates for 100 days and consider this as n=3. Each clone within this plate is considered a technical replicate. CUT&RUN samples for H3K9me3 and H3K27me3 were validated as n=2 and also by alternative methodology (DiMelo-seq). For experiments with single-cell output, the number of measured cells was set to 20000 (number of events in FACS). Immunoblots are validated at a minimum n=2 where screening for gene knockouts.
Data exclusions	No data was excluded from the analysis, with the exception of analysis involving CENP-A-containing HORs in DiMelo-seq data. Here, cases such as Cen3 (Hap1 and Hap2), Cen4 (Hap1), and Cen13 (Hap2) which contain multiple split active HORs were contained to just the CENP-A-containing HOR.The following chromosomes were not considered in all CDR analyses: Cen4 Hap2 (neocentromere-containing haplotype); CenX Hap2 (not present in genome), Cen18 Hap1 and Cen18 Hap2 (poorly-defined CDRs).This is described in full in the methods section.
Replication	Replication of results was ensured by validation by independent methodologies (CUT&RUN, Immunoblot, DiMelo-seq). e.g. validation of gene knockout effect. All experiments were replicated at least twice. The sample number and replicate number per each experiment were included in the figure legends. The sample number and replicate number per each experiment are either in the figure legends and described in text as appropriate.
Randomization	Randomization of samples was performed for all experiments. This randomization included cell growth (randomized arrangement in growth chambers and in tissue culture plates), cell collection (randomized order in processing samples) and sequencing library preparation (randomization in the usage of bardcode sets for multiplexing).
Blinding	Blinding was not relevant for the study. The study was not based on the prior assumptions on the responses to experiment treatments, nor involve clinical trials. For unbiased interpretation of data presented in the study, the results were interpreted and consulted independently by co-authors, collaborators and departmental bodies.

Reporting for specific materials, systems and methods

We require information from authors about some types of materials, experimental systems and methods used in many studies. Here, indicate whether each material, system or method listed is relevant to your study. If you are not sure if a list item applies to your research, read the appropriate section before selecting a response.

Materials & experimental systems

n/a	Involved in the study
☐	☒ Antibodies
☐	☒ Eukaryotic cell lines
☒	☐ Palaeontology and archaeology
☒	☐ Animals and other organisms
☒	☐ Clinical data
☒	☐ Dual use research of concern
☒	☐ Plants

Methods

n/a	Involved in the study
☐	☒ ChIP-seq
☐	☒ Flow cytometry
☒	☐ MRI-based neuroimaging

Antibodies

Antibodies used

SUV39H1 Cell Signalling (D11B6)
SUV39H2 Abcam (ab190870)
SUZ12 Cell Signalling (D39F6)
SETDB1 Cell Signalling (C1C12)
CENP-A Cell Signalling (2186S)
Histone H4 Merck Millipore (05-858)
DNMT3B Cell Signalling (E2Q3Z)
α-tubulin Sigma (T9026)
H3K9me3 Abcam (ab8898)
Rabbit IgG IRDye® 800CW (926-32211)
Mouse IgG Rockland DyLight™ 680 (610-744-124)
Rabbit IgG Jackson ImmunoResearch HRP (111-035-008)
CENP-A Enzo (ADI-KAM-CC006)
H3K9me3 Abcam (ab8898)
CENP-C (Human CENP-C aa1-198, Mouse IgG1k), Gift from Don Cleveland, described in Mitra et al, 2020 Nat Comms (ref 93)
Mouse IgG1 Cell Signalling (G3A1)
Rabbit IgG Cell Signalling (2729)
H3K27me3 Cell Signalling (C36B11)
Rabbit IgG EpiCypher (13-0042)

Validation

All antibodies are validated in both wild-type and mutant context for specificity. Furthermore, each antibody is supplier validated for human cells for each application (IF, Immonoblot, CUT&RUN). Antibodies for DiMelo-Seq (CENP-A Enzo ADI-KAM-CC006; H3K9me3 Abcam ab8898, H3K27me3 Cell Signalling (C36B11), CENP-C, Mouse IgG1 Cell Signalling (G3A1), Rabbit IgG Cell Signalling (2729)) were tested and validated at a range of concentrations, settling at dilutions outlined in Table 3, with 6 million diploid cells input. Target specificity CENP-A was validated with CENP-A AID degron, in addition to validation at well characterized loci for on target specificity for other targets.

Eukaryotic cell lines

Policy information about cell lines and Sex and Gender in Research

Cell line source(s)

The base cell line used in all experiments are hTERT-RPE1 cells, originally sourced from American Type Culture Collection (ATCC; CRL-4000). Neo4p13 cell lines are derived as described in Murillo Pineda et al (ref 18). AID-eYFP-CENP-A lines were derived as described in Hoffman et al (ref 53).

Authentication

These lines are authenticated by spectral karyotyping. Transgenic lines generated were all validated by western blot, in addition to CUT&RUN and/or DiMeLo-seq for well-characterised regions.

Mycoplasma contamination

These cell lines are negative for Mycoplasma contamination

Commonly misidentified lines
(See ICLAC register)

The line used in this study is not a misidentified line, according to ICLAC.

Plants

Seed stocks	n/a
Novel plant genotypes	n/a
Authentication	n/a

ChIP-seq

Data deposition

☒ Confirm that both raw and final processed data have been deposited in a public database such as GEO.

☐ Confirm that you have deposited or provided access to graph files (e.g. BED files) for the called peaks.

Data access links
May remain private before publication.

Raw sequencing data is available at European Nucleotide Archive (ENA) under accession code PRJEB85119. All processed data files (bigwig files, genome annotation bed files etc) are available on the BioStudies database under accession code S-BSST2137.

Files in database submission

ENA Files (PRJEB85119):
s6_r2.fastq.gz,s6_r1.fastq.gz,DNMT3B_SETDB1KO_MouseIgG_refmatch.bam,DNMT3BKO_MouseIgG_refmatch.bam,SETDB1KO_MouseIgG_refmatch.bam,SUV39SUZ12KO_MouseIgG_refmatch.bam,SUV392KO_MouseIgG_refmatch.bam,Parent4p13EP_MouseIgG_refmatch.bam,SETDB1KO_RabbitIgG_refmatch.bam,SUV39SUZ12KO_RabbitIgG_refmatch.bam,SUV392KO_RabbitIgG_refmatch.bam,Parent4p13EP_RabbitIgG_refmatch.bam,SETDB1KO_H3K27me3_refmatch.bam,SUV39SUZ12KO_H3K27me3_refmatch.bam,SUV392KO_H3K27me3_refmatch.bam,Parent4p13EP_H3K27me3_refmatch.bam,SETDB1KO_CENPC_refmatch.bam,SUV39SUZ12KO_CENPC_refmatch.bam,SUV392KO_CENPC_refmatch.bam,Parent4p13EP_CENPC_refmatch.bam,DNMT3B_SETDB1KO_CENPA_refmatch.bam,DNMT3BKO_CENPA_refmatch.bam,SUV392KO_CENPA_refmatch.bam,s11_r2.fastq.gz,s11_r1.fastq.gz,s10_r2.fastq.gz,s10_r1.fastq.gz,s9_r2.fastq.gz,s9_r1.fastq.gz,s8_r2.fastq.gz,s8_r1.fastq.gz,s7_r2.fastq.gz,s7_r1.fastq.gz,s6_r2.fastq.gz,s6_r1.fastq.gz,s5_r2.fastq.gz,s5_r1.fastq.gz,s4_r2.fastq.gz,s4_r1.fastq.gz,s3_r2.fastq.gz,s3_r1.fastq.gz,s2_r2.fastq.gz,s2_r1.fastq.gz,s1_r2.fastq.gz,s1_r1.fastq.gz,s9_CENPA_2KO_r2.fastq.gz,s9_CENPA_2KO_r1.fastq.gz,s7_CENPA_2KO_r2.fastq.gz,s7_CENPA_2KO_r1.fastq.gz,s5_CENPA_2KO_r2.fastq.gz,s5_CENPA_2KO_r1.fastq.gz,s2_CENPA_2KO_r2.fastq.gz,s2_CENPA_2KO_r1.fastq.gz,s6_H3K27me3_r2.fastq.gz,s6_H3K27me3_r1.fastq.gz,s3_H3K9me3_r2.fastq.gz,s3_H3K9me3_r1.fastq.gz,s5_CENPA_3KO_r2.fastq.gz,s5_CENPA_3KO_r1.fastq.gz,s8_CENPA_r2.fastq.gz,s8_CENPA_r1.fastq.gz,s6_CENPA_r2.fastq.gz,s6_CENPA_r1.fastq.gz,s4_CENPA_r2.fastq.gz,s4_CENPA_r1.fastq.gz,s5_H3K9me3_r2.fastq.gz,s5_H3K9me3_r1.fastq.gz,s5_CENPA_r2.fastq.gz,s5_CENPA_r1.fastq.gz,s3_CENPA_r2.fastq.gz,s3_CENPA_r1.fastq.gz,s10_CENPA_3KO_r2.fastq.gz,s10_CENPA_3KO_r1.fastq.gz,s8_CENPA_3KO_r2.fastq.gz,s8_CENPA_3KO_r1.fastq.gz,s6_CENPA_2KO_r2.fastq.gz,s6_CENPA_2KO_r1.fastq.gz,s4_CENPA_2KO_r2.fastq.gz,s4_CENPA_2KO_r1.fastq.gz,s13_CENPA_3KO_r2.fastq.gz,s13_CENPA_3KO_r1.fastq.gz,s11_CENPA_3KO_r2.fastq.gz,s11_CENPA_3KO_r1.fastq.gz,s9_CENPA_3KO_r2.fastq.gz,s9_CENPA_3KO_r1.fastq.gz,s7_H3K27me3_r2.fastq.gz,s7_H3K27me3_r1.fastq.gz,s8_H3K27me3_r2.fastq.gz,s8_H3K27me3_r1.fastq.gz,s2_H3K9me3_r2.fastq.gz,s2_H3K9me3_r1.fastq.gz,s10_CENPA_r2.fastq.gz,s10_CENPA_r1.fastq.gz,s3_CENPA_2KO_r2.fastq.gz,s3_CENPA_2KO_r1.fastq.gz,s2_CENPA_r2.fastq.gz,s2_CENPA_r1.fastq.gz,s7_CENPA_3KO_r2.fastq.gz,s7_CENPA_3KO_r1.fastq.gz,s1_H3K9me3_r2.fastq.gz,s1_H3K9me3_r1.fastq.gz,s14_CENPA_3KO_r2.fastq.gz,s14_CENPA_3KO_r1.fastq.gz,s12_CENPA_3KO_r2.fastq.gz,s12_CENPA_3KO_r1.fastq.gz,s5_H3K27me3_r2.fastq.gz,s5_H3K27me3_r1.fastq.gz,s9_CENPA_r2.fastq.gz,s9_CENPA_r1.fastq.gz,s7_CENPA_r2.fastq.gz,s7_CENPA_r1.fastq.gz,s10_CENPA_2KO_r2.fastq.gz,s10_CENPA_2KO_r1.fastq.gz,s8_CENPA_2KO_r2.fastq.gz,s8_CENPA_2KO_r1.fastq.gz,s9_H3K27me3_r2.fastq.gz,s9_H3K27me3_r1.fastq.gz,s1_CENPA_3KO_r2.fastq.gz,s1_CENPA_3KO_r1.fastq.gz,s1_CENPA_r2.fastq.gz,s1_CENPA_r1.fastq.gz,s4_H3K9me3_r2.fastq.gz,s4_H3K9me3_r1.fastq.gz,s2_CENPA_3KO_r2.fastq.gz,s2_CENPA_3KO_r1.fastq.gz,s11_CENPA_2KO_r2.fastq.gz,s11_CENPA_2KO_r1.fastq.gz,s1_CENPA_2KO_r2.fastq.gz,s1_CENPA_2KO_r1.fastq.gz,s15_CENPA_3KO_r2.fastq.gz,s15_CENPA_3KO_r1.fastq.gz,RPE1AIDYFPCENPA_IAA_CENPA_refmatch.bam,RPE1AIDYFPCENPA_NEG_CENPA_refmatch.bam,SUV39SUZ12KO_100d1B_CENPA_refmatch.bam,Parent4p13100d12_CENPA_refmatch.bam,SETDB1KO_CENPA_refmatch.bam,SUV39SUZ12KO_CENPA_refmatch.bam,Parent4p130gen_CENPA_refmatch.bam,SETDB1EP_H3K9me3_refmatch.bam,SUV39SUZ12KO_H3K9me3_refmatch.bam,SUV392KO_H3K9me3_refmatch.bam,Parent4p13EP_H3K9me3_refmatch.bam

BioStudies Files (S-BSST2137):
2KOEP_H3K27me3_6mA_HOR_modfraction.bw,2KOEP_H3K27me3_6mA_chr4_modfraction.bw,2KOEP_H3K27me3_CG_HOR_modfraction.bw,2KOEP_H3K27me3_CG_chr4_modfraction.bw,3KOEP_H3K27me3_6mA_HOR_modfraction.bw,3KOEP_H3K27me3_6mA_chr4_modfraction.bw,3KOEP_H3K27me3_CG_HOR_modfraction.bw,3KOEP_H3K27me3_CG_chr4_modfraction.bw,4p130gen_H3K27me3_6mA_HOR_modfraction.bw,4p130gen_H3K27me3_6mA_chr4_modfraction.bw,4p130gen_H3K27me3_CG_HOR_modfraction.bw,4p130gen_H3K27me3_CG_chr4_modfraction.bw,SETDB1KO_H3K27me3_6mA_HOR_modfraction.bw,SETDB1KO_H3K27me3_6mA_chr4_modfraction.bw,SETDB1KO_H3K27me3_CG_HOR_modfraction.bw,SETDB1KO_H3K27me3_CG_chr4_modfraction.bw,2KOEP_H3K27me3_CG.bed,2KOEP_H3K27me3_m6a.bed,3KOEP_H3K27me3_CG.bed,3KOEP_H3K27me3_m6a.bed,4p130gen_H3K27me3_CG.bed,4p130gen_H3K27me3_m6a.bed,SETDB1KO_H3K27me3_CG.bed,SETDB1KO_H3K27me3_m6a.bed,2KOEP_H3K27me3_6mA_HOR.tsv,2KOEP_H3K27me3_6mA_chr4.tsv,2KOEP_H3K27me3_CG_HOR.tsv,2KOEP_H3K27me3_CG_chr4.tsv,3KOEP_H3K27me3_6mA_HOR.tsv,3KOEP_H3K27me3_6mA_chr4.tsv,3KOEP_H3K27me3_CG_HOR.tsv,3KOEP_H3K27me3_CG_chr4.tsv,4p130gen_H3K27me3_6mA_HOR.tsv,4p130gen_H3K27me3_6mA_chr

4.tsv,4p130gen_H3K27me3_CG_HOR.tsv,4p130gen_H3K27me3_CG_chr4.tsv,SETDB1KO_H3K27me3_6mA_HOR.tsv,SETDB1KO_H3K27me3_6mA_chr4.tsv,SETDB1KO_H3K27me3_CG_HOR.tsv,SETDB1KO_H3K27me3_CG_chr4.tsv,RPE1_active_HORs_CENPAcontaining.bed,RPE1_active_HORs_CENPAcontaining_nochr4hap2.bed,RPE1_active_HORs_CENPAcontaining_nochr4hap2_sorted.bed,RPE1_active_HORs_filtered.bed,4p13_CDRboundaries_hap1_hap2.bed,AS-HOR+SF-vs-hap1.for.syri.bed,AS-HORvsRPE1.chrhap2.bed,RPE1_active_HORs.bed,RPE1_divergent_HORs.bed,RPE1_inactive_HORs.bed,2KO_CDR_manual_merged.bed,3KO100d1B_CDR_manual_merged.bed,3KO_CDR_manual_merged.bed,4p13100d12_CDR_manual_merged.bed,DNMT3B_CDR_manual_merged.bed,Neo4p13_CDR_manual_merged.bed,SETDB1_CDR_manual_merged.bed,SETDB1_DNMT3B_CDR_manual_merged.bed,2KO_primaryCDRs.bed,3KO100d1B_primaryCDRs.bed,3KO_primaryCDRs.bed,4p13100d12_primaryCDRs.bed,DNMT3B_primaryCDRs.bed,Neo4p13_primaryCDRs.bed,SETDB1_DNMT3B_primaryCDRs.bed,SETDB1_primaryCDRs.bed,2KOEP_H3K9me3_6mA_activeHOR_modfraction.bw,2KOEP_H3K9me3_6mA_chr4_modfraction.bw,2KOEP_H3K9me3_CG_activeHOR_mod_fraction.bw,2KOEP_H3K9me3_CG_chr4_modfraction.bw,3KOEP_H3K9me3_6mA_activeHOR_modfraction.bw,3KOEP_H3K9me3_6mA_chr4_modfraction.bw,3KOEP_H3K9me3_CG_activeHOR_mod_fraction.bw,3KOEP_H3K9me3_CG_chr4_modfraction.bw,4p13EP_H3K9me3_6mA_activeHOR_modfraction.bw,4p13EP_H3K9me3_6mA_chr4_modfraction.bw,4p13EP_H3K9me3_CG_activeHOR_mod_fraction.bw,4p13EP_H3K9me3_CG_chr4_modfraction.bw,SDB1EP_H3K9me3_6mA_activeHOR_modfraction.bw,SDB1EP_H3K9me3_6mA_chr4_modfraction.bw,SDB1EP_H3K9me3_CG_activeHOR_mod_fraction.bw,SDB1EP_H3K9me3_CG_chr4_modfraction.bw,2KOEP_H3K9me3_CG.bed.gz,2KOEP_H3K9me3_m6a.bed.gz,3KOEP_H3K9me3_CG.bed.gz,3KOEP_H3K9me3_m6a.bed.gz,4p13EP_H3K9me3_CG.bed.gz,4p13EP_H3K9me3_m6a.bed.gz,SDB1EP_H3K9me3_CG.bed.gz,SDB1EP_H3K9me3_m6a.bed.gz,2KOEP_H3K9me3_6mA_activeHOR.tsv,2KOEP_H3K9me3_6mA_chr4.tsv,2KOEP_H3K9me3_CG_activeHOR.tsv,2KOEP_H3K9me3_CG_chr4.tsv,3KOEP_H3K9me3_6mA_activeHOR.tsv,3KOEP_H3K9me3_6mA_chr4.tsv,3KOEP_H3K9me3_CG_activeHOR.tsv,3KOEP_H3K9me3_CG_chr4.tsv,4p13EP_H3K9me3_6mA_activeHOR.tsv,4p13EP_H3K9me3_6mA_chr4.tsv,4p13EP_H3K9me3_CG_activeHOR.tsv,4p13EP_H3K9me3_CG_chr4.tsv,SDB1EP_H3K9me3_6mA_activeHOR.tsv,SDB1EP_H3K9me3_6mA_chr4.tsv,SDB1EP_H3K9me3_CG_activeHOR.tsv,SDB1EP_H3K9me3_CG_chr4.tsv,2KOEPmono17_CENPA_6mA_activeHORmod_fraction.bw,2KOEPmono17_CENPA_6mA_chr4_modfraction.bw,2KOEPmono17_CENPA_CG_chr4_modfraction.bw,3BSDB1KO_CENPA_6mA_activeHORmod_fraction.bw,3BSDB1KO_CENPA_6mA_chr4_modfraction.bw,3BSDB1KO_CENPA_CG_chr4_modfraction.bw,3KO100d1B_CENPA_6mA_activeHORmod_fraction.bw,3KO100d1B_CENPA_6mA_chr4_modfraction.bw,3KO100d1B_CENPA_CG_activeHOR_mod_fraction.bw,3KO100d1B_CENPA_CG_chr4_modfraction.bw,3KOEPmono14_CENPA_6mA_activeHORmod_fraction.bw,3KOEPmono14_CENPA_6mA_chr4_modfraction.bw,3KOEPmono14_CENPA_CG_activeHOR_mod_fraction.bw,3KOEPmono14_CENPA_CG_chr4_modfraction.bw,4p130gen_CENPA_6mA_activeHORmod_fraction.bw,4p130gen_CENPA_6mA_chr4_modfraction.bw,4p130gen_CENPA_CG_activeHOR_mod_fraction.bw,4p130gen_CENPA_CG_chr4_modfraction.bw,4p13100d12_CENPA_6mA_activeHORmod_fraction.bw,4p13100d12_CENPA_6mA_chr4_modfraction.bw,4p13100d12_CENPA_CG_activeHOR_mod_fraction.bw,4p13100d12_CENPA_CG_chr4_modfraction.bw,DNMT3BKO_CENPA_6mA_activeHORmod_fraction.bw,DNMT3BKO_CENPA_6mA_chr4_modfraction.bw,DNMT3BKO_CENPA_CG_chr4_modfraction.bw,RPE1AIDYFPCENPA_IAA_CENPA_6mA_activeHORmod_fraction.bw,RPE1AIDYFPCENPA_IAA_CENPA_6mA_chr4_modfraction.bw,RPE1AIDYFPCENPA_IAA_CENPA_CG_activeHOR_mod_fraction.bw,RPE1AIDYFPCENPA_IAA_CENPA_CG_chr4_modfraction.bw,RPE1AIDYFPCENPA_neg_CENPA_6mA_activeHORmod_fraction.bw,RPE1AIDYFPCENPA_neg_CENPA_6mA_chr4_modfraction.bw,RPE1AIDYFPCENPA_neg_CENPA_CG_activeHOR_mod_fraction.bw,RPE1AIDYFPCENPA_neg_CENPA_CG_chr4_modfraction.bw,SDB1KO_CENPA_6mA_activeHORmod_fraction.bw,SDB1KO_CENPA_6mA_chr4_modfraction.bw,SDB1KO_CENPA_CG_activeHOR_mod_fraction.bw,SDB1KO_CENPA_CG_chr4_modfraction.bw,2KOEPmono17_CENPA_CG.bed,2KOEPmono17_CENPA_m6a.bed,3BSDB1KO_CENPA_CG.bed,3BSDB1KO_CENPA_m6a.bed,3KO100d1B_CENPA_CG.bed.gz,3KO100d1B_CENPA_m6a.bed.gz,3KOEPmono14_CENPA_CG.bed.gz,3KOEPmono14_CENPA_m6a.bed.gz,4p130gen_CENPA_CG.bed.gz,4p130gen_CENPA_m6a.bed.gz,4p13100d12_CENPA_CG.bed.gz,4p13100d12_CENPA_m6a.bed.gz,DNMT3BKO_CENPA_CG.bed,DNMT3BKO_CENPA_m6a.bed,RPE1AIDYFPCENPA_IAA_CENPA_CG.bed.gz,RPE1AIDYFPCENPA_IAA_CENPA_m6a.bed.gz,RPE1AIDYFPCENPA_neg_CENPA_CG.bed.gz,RPE1AIDYFPCENPA_neg_CENPA_m6a.bed.gz,SDB1KO_CENPA_CG.bed.gz,SDB1KO_CENPA_m6a.bed.gz,2KOEPmono17_CENPA_6mA_activeHOR.tsv,2KOEPmono17_CENPA_6mA_chr4.tsv,2KOEPmono17_CENPA_CG_activeHOR.tsv,2KOEPmono17_CENPA_CG_chr4.tsv,3BSDB1KO_CENPA_6mA_activeHOR.tsv,3BSDB1KO_CENPA_6mA_chr4.tsv,3BSDB1KO_CENPA_CG_activeHOR.tsv,3BSDB1KO_CENPA_CG_chr4.tsv,3KO100d1B_CENPA_6mA_activeHOR.tsv,3KO100d1B_CENPA_6mA_chr4.tsv,3KO100d1B_CENPA_CG_activeHOR.tsv,3KO100d1B_CENPA_CG_chr4.tsv,3KOEPmono14_CENPA_6mA_activeHOR.tsv,3KOEPmono14_CENPA_6mA_chr4.tsv,3KOEPmono14_CENPA_CG_activeHOR.tsv,3KOEPmono14_CENPA_CG_chr4.tsv,4p130gen_CENPA_6mA_activeHOR.tsv,4p130gen_CENPA_6mA_chr4.tsv,4p130gen_CENPA_CG_activeHOR.tsv,4p130gen_CENPA_CG_chr4.tsv,4p13100d12_CENPA_6mA_activeHOR.tsv,4p13100d12_CENPA_6mA_chr4.tsv,4p13100d12_CENPA_CG_activeHOR.tsv,4p13100d12_CENPA_CG_chr4.tsv,DNMT3BKO_CENPA_6mA_activeHOR.tsv,DNMT3BKO_CENPA_6mA_chr4.tsv,DNMT3BKO_CENPA_CG_chr4.tsv,RPE1AIDYFPCENPA_IAA_CENPA_6mA_activeHOR.tsv,RPE1AIDYFPCENPA_IAA_CENPA_6mA_chr4.tsv,RPE1AIDYFPCENPA_IAA_CENPA_CG_activeHOR.tsv,RPE1AIDYFPCENPA_IAA_CENPA_CG_chr4.tsv,RPE1AIDYFPCENPA_neg_CENPA_6mA_activeHOR.tsv,RPE1AIDYFPCENPA_neg_CENPA_6mA_chr4.tsv,RPE1AIDYFPCENPA_neg_CENPA_CG_activeHOR.tsv,RPE1AIDYFPCENPA_neg_CENPA_CG_chr4.tsv,SDB1KO_CENPA_6mA_activeHOR.tsv,SDB1KO_CENPA_6mA_chr4.tsv,SDB1KO_CENPA_CG_activeHOR.tsv,SDB1KO_CENPA_CG_chr4.tsv,SampleID_SUV39H1H2_100d_CENPA_cutrun.xlsx,s10_rmvdups_2KO_CENPA.bw,s11_rmvdups_2KO_CENPA.bw,s1_rmvdups_2KO_CENPA.bw,s2_rmvdups_2KO_CENPA.bw,s3_rmvdups_2KO_CENPA.bw,s4_rmvdups_2KO_CENPA.bw,s5_rmvdups_2KO_CENPA.bw,s6_rmvdups_2KO_CENPA.bw,s7_rmvdups_2KO_CENPA.bw,s8_rmvdups_2KO_CENPA.bw,s9_rmvdups_2KO_CENPA.bw,SampleID_SETDB1KO_100d_CENPA.xlsx,s10_rmvdups_CENPA.bw,s11_rmvdups_CENPA.bw,s1_rmvdups_CENPA.bw,s2_rmvdups_CENPA.bw,s3_rmvdups_CENPA.bw,s4_rmvdups_CENPA.bw,s5_rmvdups_CENPA.bw,s6_rmvdups_CENPA.bw,s7_rmvdups_CENPA.bw,s8_rmvdups_CENPA.bw,s9_rmvdups_CENPA.bw,2KOEP_MouseIgG_6mA_HOR_modfraction.bw,2KOEP_MouseIgG_6mA_chr4_modfraction.bw,2KOEP_MouseIgG_CG_HOR_modfraction.bw,2KOEP_MouseIgG_CG_chr4_modfraction.bw,3BSETDB1KO_MouseIgG_6mA_HOR_modfraction.bw,3BSETDB1KO_MouseIgG_6mA_chr4_modfraction.bw,3BSETDB1KO_MouseIgG_CG_HOR_modfraction.bw,3BSETDB1KO_MouseIgG_CG_chr4_modfraction.bw,3KOEP_MouseIgG_6mA_HOR_modfraction.bw,3KOEP_MouseIgG_6mA_chr4_modfraction.bw,3KOEP_MouseIgG_CG_HOR_modfraction.bw,3KOEP_MouseIgG_CG_chr4_modfraction.bw,4p130gen_MouseIgG_6mA_HOR_modfraction.bw,4p130gen_MouseIgG_6mA_chr4_modfraction.bw,4p130gen_MouseIgG_CG_HOR_modfraction.bw,4p130gen_MouseIgG_CG_chr4_modfraction.bw,DNMT3BKO_MouseIgG_6mA_HOR_modfraction.bw,DNMT3BKO_MouseIgG_6mA_chr4_modfraction.bw,DNMT3BKO_MouseIgG_CG_HOR_modfraction.bw,DNMT3BKO_MouseIgG_CG_chr4_modfraction.bw,SETDB1KO_MouseIgG_6mA_HOR_modfraction.bw,SETDB1KO_MouseIgG_6mA_chr4_modfraction.bw,SETDB1KO_MouseIgG_CG_HOR_modfraction.bw,SETDB1KO_MouseIgG_CG_chr4_modfraction.bw,2KOEP_MouseIgG_CG.bed,2KOEP_MouseIgG_m6a.bed,3BSETDB1KO_MouseIgG_CG.bed,3BSETDB1KO_MouseIgG_m6a.bed,3KOEP_MouseIgG_CG.bed,3KOEP_MouseIgG_m6a.bed,4p130gen_MouseIgG_CG.bed,4p130gen_MouseIgG_m6a.bed,DNMT3BKO_MouseIgG_CG.bed,DNMT3BKO_MouseIgG_m6a.bed,SETDB1KO_MouseIgG_CG.bed,SETDB1KO_MouseIgG_m6a.bed,2KOEP_MouseIgG_6mA_HOR.tsv,2KOEP_MouseIgG_6mA_chr4.tsv,2KOEP_MouseIgG_CG_HOR.tsv,2KOEP_MouseIgG_CG_chr4.tsv,3BSETDB1KO_MouseIgG_6mA_HOR.tsv,3BSETDB1KO_MouseIgG_6mA_chr4.tsv,3B

SETDB1KO_MouseIgG_CG_HOR.tsv,3BSETDB1KO_MouseIgG_CG_chr4.tsv,3KOEP_MouseIgG_6mA_HOR.tsv,3KOEP_MouseIgG_6mA_chr4.tsv,3KOEP_MouseIgG_CG_HOR.tsv,3KOEP_MouseIgG_CG_chr4.tsv,4p130gen_MouseIgG_6mA_HOR.tsv,4p130gen_MouseIgG_6mA_chr4.tsv,4p130gen_MouseIgG_CG_HOR.tsv,4p130gen_MouseIgG_CG_chr4.tsv,DNMT3BKO_MouseIgG_6mA_HOR.tsv,DNMT3BKO_MouseIgG_6mA_chr4.tsv,DNMT3BKO_MouseIgG_CG_HOR.tsv,DNMT3BKO_MouseIgG_CG_chr4.tsv,SETDB1KO_MouseIgG_6mA_HOR.tsv,SETDB1KO_MouseIgG_6mA_chr4.tsv,SETDB1KO_MouseIgG_CG_HOR.tsv,SETDB1KO_MouseIgG_CG_chr4.tsv,SampleID_H3K9me3_cutrun.xlsx,s1_rmvdups_H3K9me3.bw,s2_rmvdups_H3K9me3.bw,s3_rmvdups_H3K9me3.bw,s4_rmvdups_H3K9me3.bw,s5_rmvdups_H3K9me3.bw,s6_rmvdups_H3K9me3.bw,SampleID_H3K27me3_cutrun.xlsx,s5_rmvdups_H3K27me3.bw,s6_rmvdups_H3K27me3.bw,s7_rmvdups_H3K27me3.bw,s8_rmvdups_H3K27me3.bw,s9_rmvdups_H3K27me3.bw,2KOEP_CENPC_6mA_HOR_modfraction.bw,2KOEP_CENPC_6mA_chr4_modfraction.bw,2KOEP_CENPC_CG_HOR_modfraction.bw,3KOEP_CENPC_6mA_HOR_modfraction.bw,3KOEP_CENPC_6mA_chr4_modfraction.bw,3KOEP_CENPC_CG_HOR_modfraction.bw,4p130gen_CENPC_6mA_HOR_modfraction.bw,4p130gen_CENPC_6mA_chr4_modfraction.bw,4p130gen_CENPC_CG_HOR_modfraction.bw,SETDB1KO_CENPC_6mA_HOR_modfraction.bw,SETDB1KO_CENPC_6mA_chr4_modfraction.bw,SETDB1KO_CENPC_CG_HOR_modfraction.bw,2KOEP_CENPC_CG.bed,2KOEP_CENPC_m6a.bed,3KOEP_CENPC_CG.bed,3KOEP_CENPC_m6a.bed,4p130gen_CENPC_CG.bed,4p130gen_CENPC_m6a.bed,SETDB1KO_CENPC_CG.bed,SETDB1KO_CENPC_m6a.bed,2KOEP_CENPC_6mA_HOR.tsv,2KOEP_CENPC_6mA_chr4.tsv,2KOEP_CENPC_CG_HOR.tsv,2KOEP_CENPC_CG_chr4.tsv,3KOEP_CENPC_6mA_HOR.tsv,3KOEP_CENPC_6mA_chr4.tsv,3KOEP_CENPC_CG_HOR.tsv,3KOEP_CENPC_CG_chr4.tsv,4p130gen_CENPC_6mA_HOR.tsv,4p130gen_CENPC_6mA_chr4.tsv,4p130gen_CENPC_CG_HOR.tsv,4p130gen_CENPC_CG_chr4.tsv,SETDB1KO_CENPC_6mA_HOR.tsv,SETDB1KO_CENPC_6mA_chr4.tsv,SETDB1KO_CENPC_CG_HOR.tsv,SETDB1KO_CENPC_CG_chr4.tsv,SampleID_4p13100d_CENPA_cutrun.xlsx,s10_rmvdups_CENPA.bw,s1_rmvdups_CENPA.bw,s2_rmvdups_CENPA.bw,s3_rmvdups_CENPA.bw,s4_rmvdups_CENPA.bw,s5_rmvdups_CENPA.bw,s6_rmvdups_CENPA.bw,s7_rmvdups_CENPA.bw,s8_rmvdups_CENPA.bw,s9_rmvdups_CENPA.bw,2KOEP_RabbitIgG_6mA_HOR_modfraction.bw,2KOEP_RabbitIgG_6mA_chr4_modfraction.bw,2KOEP_RabbitIgG_CG_HOR_modfraction.bw,3KOEP_RabbitIgG_6mA_HOR_modfraction.bw,3KOEP_RabbitIgG_6mA_chr4_modfraction.bw,3KOEP_RabbitIgG_CG_HOR_modfraction.bw,4p130gen_RabbitIgG_6mA_HOR_modfraction.bw,4p130gen_RabbitIgG_6mA_chr4_modfraction.bw,4p130gen_RabbitIgG_CG_HOR_modfraction.bw,SETDB1KO_RabbitIgG_6mA_HOR_modfraction.bw,SETDB1KO_RabbitIgG_6mA_chr4_modfraction.bw,SETDB1KO_RabbitIgG_CG_HOR_modfraction.bw,2KOEP_RabbitIgG_CG.bed,2KOEP_RabbitIgG_m6a.bed,3KOEP_RabbitIgG_CG.bed,3KOEP_RabbitIgG_m6a.bed,4p130gen_RabbitIgG_CG.bed,4p130gen_RabbitIgG_m6a.bed,SETDB1KO_RabbitIgG_CG.bed,SETDB1KO_RabbitIgG_m6a.bed,2KOEP_RabbitIgG_6mA_HOR.tsv,2KOEP_RabbitIgG_6mA_chr4.tsv,2KOEP_RabbitIgG_CG_HOR.tsv,2KOEP_RabbitIgG_CG_chr4.tsv,3KOEP_RabbitIgG_6mA_HOR.tsv,3KOEP_RabbitIgG_6mA_chr4.tsv,3KOEP_RabbitIgG_CG_HOR.tsv,3KOEP_RabbitIgG_CG_chr4.tsv,4p130gen_RabbitIgG_6mA_HOR.tsv,4p130gen_RabbitIgG_6mA_chr4.tsv,4p130gen_RabbitIgG_CG_HOR.tsv,4p130gen_RabbitIgG_CG_chr4.tsv,SETDB1KO_RabbitIgG_6mA_HOR.tsv,SETDB1KO_RabbitIgG_6mA_chr4.tsv,SETDB1KO_RabbitIgG_CG_HOR.tsv,SETDB1KO_RabbitIgG_CG_chr4.tsv,SampleID_SUV39H1H2SUZ12KO_100d_CENPA_cutrun.xlsx,s10_rmvdups_3KO_CENPA.bw,s11_rmvdups_3KO_CENPA.bw,s12_rmvdups_3KO_CENPA.bw,s13_rmvdups_3KO_CENPA.bw,s14_rmvdups_3KO_CENPA.bw,s15_rmvdups_3KO_CENPA.bw,s1_rmvdups_3KO_CENPA.bw,s2_rmvdups_3KO_CENPA.bw,s5_rmvdups_3KO_CENPA.bw,s7_rmvdups_3KO_CENPA.bw,s8_rmvdups_3KO_CENPA.bw,s9_rmvdups_3KO_CENPA.bw,renamehap2_convert2bw.sh,s1_PDNC4_log2CENPAoverInput.bw,s1_PDNC4_log2CENPAoverInput_rename.bw,s3_MS4221_log2CENPAoverInput.bw,s3_MS4221_log2CENPAoverInput_rename.bw,s5_IMS13q_log2CENPAoverInput.bw,s5_IMS13q_log2CENPAoverInput_rename.bw,IMS13q_LINE_Count_Violin_Plot.png,IMS13q_LTR_Count_Violin_Plot.png,IMS13q_SINE_Count_Violin_Plot.png,enrichment_data_chr13_hap2.csv,MS4221_LINE_Count_Violin_Plot.png,MS4221_LTR_Count_Violin_Plot.png,MS4221_SINE_Count_Violin_Plot.png,enrichment_data_chr8_hap2.csv,._Neo4p13_LINE_Count_Violin_Plot.png,._Neo4p13_LTR_Count_Violin_Plot.png,._enrichment_data_chr4_hap2.csv,Neo4p13_LINE_Count_Violin_Plot.png,Neo4p13_LTR_Count_Violin_Plot.png,Neo4p13_SINE_Count_Violin_Plot.png,enrichment_data_chr4_hap2.csv,PDNC4_LINE_Count_Violin_Plot.png,PDNC4_LTR_Count_Violin_Plot.png,PDNC4_SINE_Count_Violin_Plot.png,enrichment_data_chr4_hap2.csv,chr10_hap1.gff,chr11_hap1.gff,chr12_hap1.gff,chr13_hap1.gff,chr14_hap1.gff,chr15_hap1.gff,chr16_hap1.gff,chr17_hap1.gff,chr18_hap1.gff,chr19_hap1.gff,chr1_hap1.gff,chr20_hap1.gff,chr21_hap1.gff,chr22_hap1.gff,chr2_hap1.gff,chr3_hap1.gff,chr4_hap1.gff,chr5_hap1.gff,chr6_hap1.gff,chr7_hap1.gff,chr8_hap1.gff,chr9_hap1.gff,chrX_hap1.gff,chr10_LINE_hap2.bed,chr10_LTR_hap2.bed,chr10_SINE_hap2.bed,chr11_LINE_hap2.bed,chr11_LTR_hap2.bed,chr11_SINE_hap2.bed,chr12_LINE_hap2.bed,chr12_LTR_hap2.bed,chr12_SINE_hap2.bed,chr13_LINE_hap2.bed,chr13_LTR_hap2.bed,chr13_SINE_hap2.bed,chr14_LINE_hap2.bed,chr14_LTR_hap2.bed,chr14_SINE_hap2.bed,chr15_LINE_hap2.bed,chr15_LTR_hap2.bed,chr15_SINE_hap2.bed,chr16_LINE_hap2.bed,chr16_LTR_hap2.bed,chr16_SINE_hap2.bed,chr17_LINE_hap2.bed,chr17_LTR_hap2.bed,chr17_SINE_hap2.bed,chr18_LINE_hap2.bed,chr18_LTR_hap2.bed,chr18_SINE_hap2.bed,chr19_LINE_hap2.bed,chr19_LTR_hap2.bed,chr19_SINE_hap2.bed,chr1_LINE_hap2.bed,chr1_LTR_hap2.bed,chr1_SINE_hap2.bed,chr20_LINE_hap2.bed,chr20_LTR_hap2.bed,chr20_SINE_hap2.bed,chr21_LINE_hap2.bed,chr21_LTR_hap2.bed,chr21_SINE_hap2.bed,chr22_LINE_hap2.bed,chr22_LTR_hap2.bed,chr22_SINE_hap2.bed,chr2_LINE_hap2.bed,chr2_LTR_hap2.bed,chr2_SINE_hap2.bed,chr3_LINE_hap2.bed,chr3_LTR_hap2.bed,chr3_SINE_hap2.bed,chr4_LINE_hap2.bed,chr4_LTR_hap2.bed,chr4_SINE_hap2.bed,chr5_LINE_hap2.bed,chr5_LTR_hap2.bed,chr5_SINE_hap2.bed,chr6_LINE_hap2.bed,chr6_LTR_hap2.bed,chr6_SINE_hap2.bed,chr7_LINE_hap2.bed,chr7_LTR_hap2.bed,chr7_SINE_hap2.bed,chr8_LINE_hap2.bed,chr8_LTR_hap2.bed,chr8_SINE_hap2.bed,chr9_LINE_hap2.bed,chr9_LTR_hap2.bed,chr9_SINE_hap2.bed,chrX_LINE_hap2.bed,chrX_LTR_hap2.bed,chrX_SINE_hap2.bed,chr10_hap2.gff,chr11_hap2.gff,chr12_hap2.gff,chr13_hap2.gff,chr14_hap2.gff,chr15_hap2.gff,chr16_hap2.gff,chr17_hap2.gff,chr18_hap2.gff,chr19_hap2.gff,chr1_hap2.gff,chr20_hap2.gff,chr21_hap2.gff,chr22_hap2.gff,chr2_hap2.gff,chr3_hap2.gff,chr4_hap2.gff,chr5_hap2.gff,chr6_hap2.gff,chr7_hap2.gff,chr8_hap2.gff,chr9_hap2.gff,chrX_hap2.gff,TE_Analysis_IMS13q.ipynb,TE_Analysis_MS4221.ipynb,TE_Analysis_Neo4p13.ipynb,TE_Analysis_PDNC4.ipynb,TEfilter.py

Genome browser session
(e.g. UCSC)

n/a

Methodology

Replicates

CENP-A evolution experiments were designed such that a single clonal Neo4p13 line was cultured in 3 separate plates for 100 days. These 3 populations were then treated as individual biological replicates, of which we sequenced 3 monoclonal lines from. We visualize these both individually and as pooled samples with +/- variance (standard deviation). H3K9me3 and H3K27me3 were validated in duplicate (n=2). For visualization of heterochromatin at Neo4p13 (per Fig 3C), a single representative replicate per condition was chosen.

Sequencing depth

Sequencing was performed Illumina NextSeq 500 as pair-end short-read sequencing with 35bp read length (2x 35bp) and High-

Sequencing depth	Output (Cut&Run). The sequencing depth per sample across all experiments and samples was within 8-20 million uniquely mapped reads per sample.
Antibodies	The antibodies used for CUT&RUN were: H3K9me3 Abcam (ab8898), H3K27me3 Cell Signalling (C36B11), CENP-A Enzo (ADI-KAM-CC006). For DiMelo-Seq, CENP-A Enzo ADI-KAM-CC006; H3K9me3 Abcam ab8898, H3K27me3 Cell Signalling (C36B11), CENP-C (homemade), Mouse IgG1 Cell Signalling (G3A1), Rabbit IgG Cell Signalling (2729) were used.
Peak calling parameters	Peak calling was not performed. More relevant for the study was read counting for specified genomic regions i.e. centromeres/ neocentromeres. Read counting and cross-comparison between samples was performed with Deeptools v2 (multibigwig summary and bamcoverage commands).
Data quality	Data quality for CUT&RUN was assessed using Basespace and FastQC. Data quality for nanopore reads were assessed via MinKnow software.
Software	Data analysis for CUT&RUN was performed with unix-based tools: FastQC v0.12.1, Bowtie2 v2.5.1, Samtools v1.17, Picard v2.27, Deeptools v3.5.5. Nanopore sequencing analysis was performed with Dorado v7.3, modkit v0.4.0, fibertools-rs v0.5.4 and CDR-Finder v1.0.0.

Flow Cytometry

Plots

Confirm that:

☒ The axis labels state the marker and fluorochrome used (e.g. CD4-FITC).

☒ The axis scales are clearly visible. Include numbers along axes only for bottom left plot of group (a 'group' is an analysis of identical markers).

☒ All plots are contour plots with outliers or pseudocolor plots.

☒ A numerical value for number of cells or percentage (with statistics) is provided.

Methodology

Sample preparation	For cell cycle analysis based on DNA content, each reported cell line was harvested and washed with PBS. Fixation was performed by adding 1 mL of cold 70% ethanol dropwise to the cell pellet while vortexing to ensure thorough fixation and minimize clumping. Cells were fixed for at least 30 mins on ice. Fixed cells were washed twice with PBS. To selectively stain DNA, cells were treated with 50 µL of RNase A, followed by the addition of 400 µL propidium iodide (PI) solution (50 µg/ml) per million cells directly to the RNase A-treated pellet. The samples were mixed thoroughly and incubated at room temperature for 10 mins. The stained samples were analysed by flow cytometry and data was collected from at least 20,000 single cells per condition, analysed on a CytoFLEX LX Flow Cytometer (Beckman Coulter).<br><br>For fluorescence-activated cell sorting (FACS), cells were collected by centrifugation for 5 min at 500 g, re-suspended in ice-cold Sorting Medium (1% Fetal Bovine Serum in PBS, 0.25mg/mL Fungizone (Thermo Fisher Scientific), 0.25µg/mL/10µg/mL Amphotericin B/Gentamicin (GIBCO)) and filtered using 5 mL polystyrene round-bottom tubes with cell-strainer caps (Falcon) before sorting and cytometry on FACSAria III or FACSAria Fusion Cell Sorter (BD Biosciences). For sorting, the cells were collected in 96-well plates with Conditional Medium (1:1 mixture of fresh complete medium and medium collected from proliferating cell cultures that is 0.45µm filtered, supplemented with 20% Fetal Bovine Serum, 0.25mg/mL Fungizone (Thermo Fisher Scientific), 0.25µg/mL/10µg/mL Amphotericin B/Gentamicin (GIBCO)).
Instrument	FACS: ACSAria III Cell Sorter (BD Biosciences)/ACSAria Fusion Cell Sorter (BD Biosciences)<br>Flow Cytometry: CytoFLEX LX Flow Cytometer (Beckman Coulter)
Software	Data was analyzed using FlowJo™ v10.8 Software (BD Life Sciences).
Cell population abundance	All cells analysed are clonally sorted transgenic cell lines. Hence, the abundance of cells are at 100%. We solely analyse the cell cycle distrubytion and ploidy based on DNA content.
Gating strategy	Gating conditions for DNA content profiling were adjusted for Neo4p13 (control) cells and kept the same across all samples thereafter.<br><br>For single cell sorting/FACS, gating conditions were adjusted separately in each FACS experiment by estimation of cell doublet size and cross-comparison of samples between positive and negative controls.

☐ Tick this box to confirm that a figure exemplifying the gating strategy is provided in the Supplementary Information.
