# Supplementary figures and images for: Heterochromatin boundaries maintain centromere position, size and number
Source: Nat Struct Mol Biol. 2025 Nov 25;33(2):220–34. doi: 10.1038/s41594-025-01706-2 (PMC7618434; doi:10.1038/s41594-025-01706-2)

Source Data for Fig 3b (uncropped Western blots)

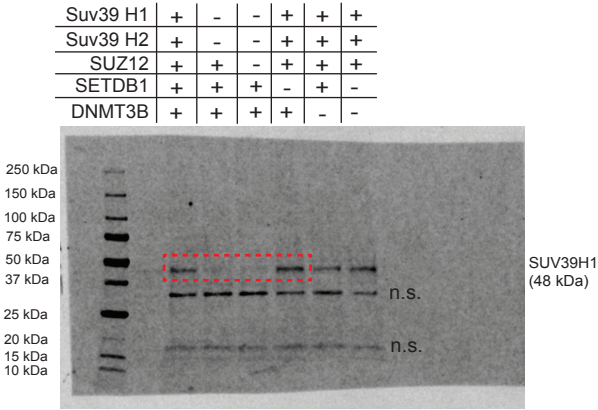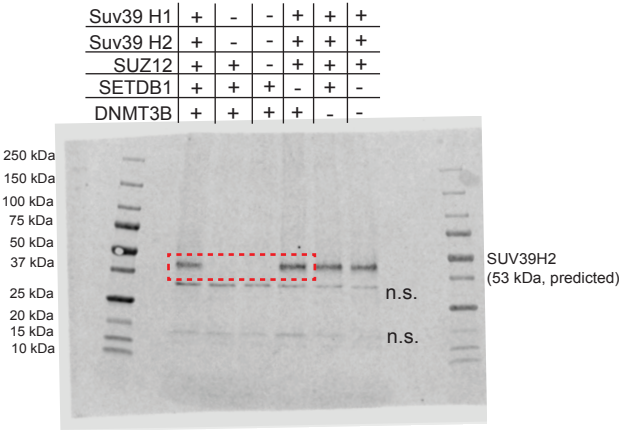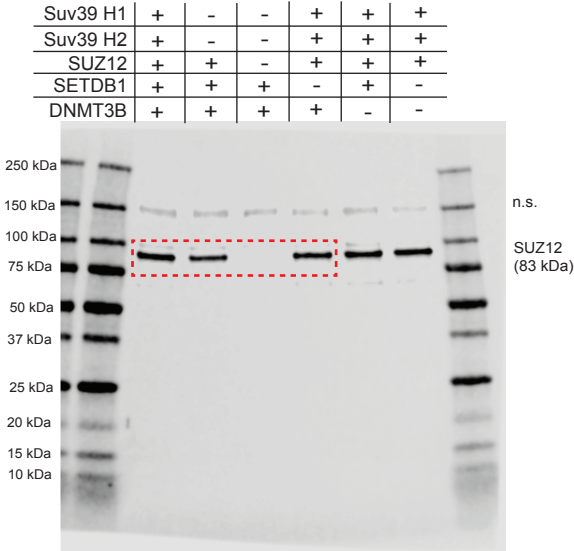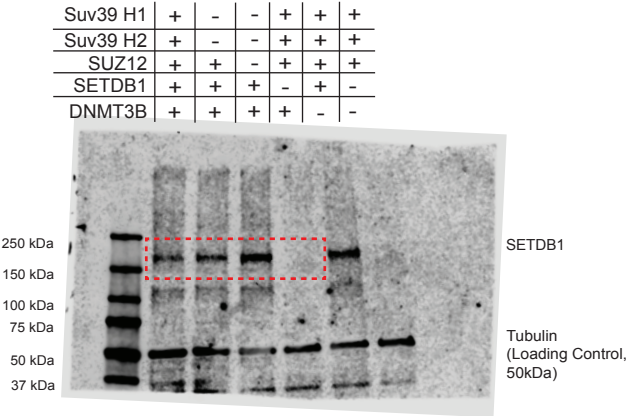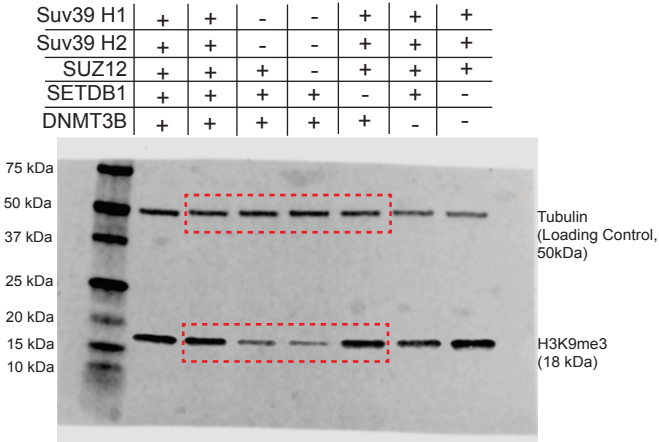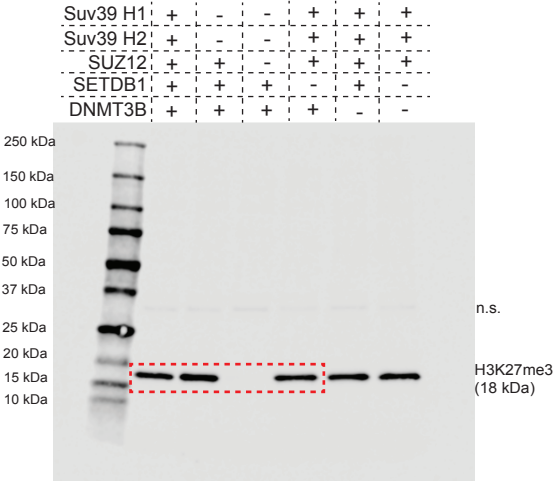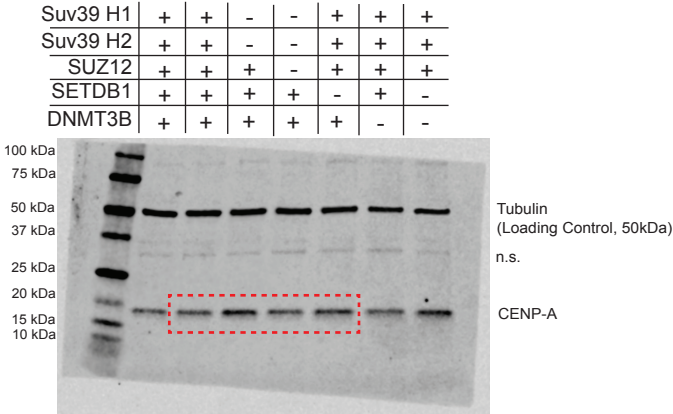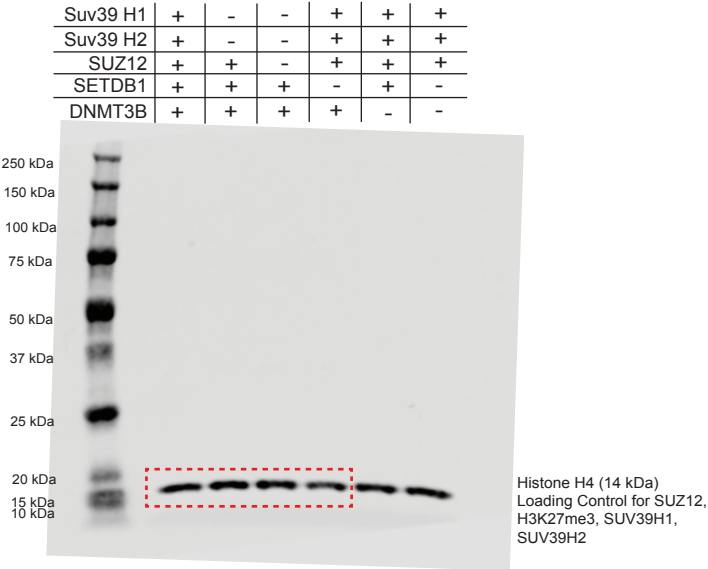

Supplement: Supplementary file 5 — Uncropped blots. [file 41594_2025_1706_MOESM5_ESM.pdf]

Source Data for Extended data Fig 2b (uncropped Western blots)

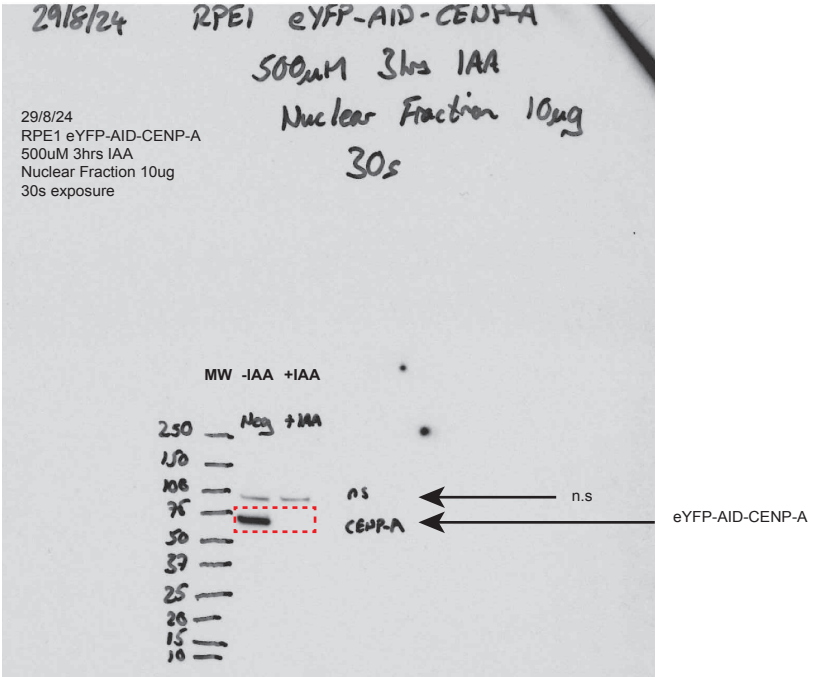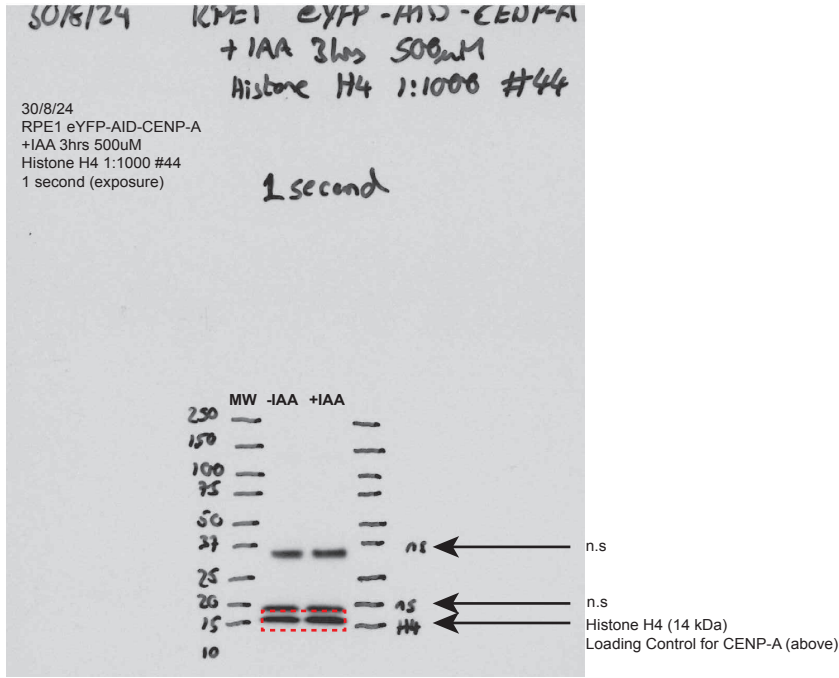

Supplement: Supplementary file 7 — Uncropped blots. [file 41594_2025_1706_MOESM7_ESM.pdf]

Source Data for Extended data Fig 7a (uncropped Western blots)

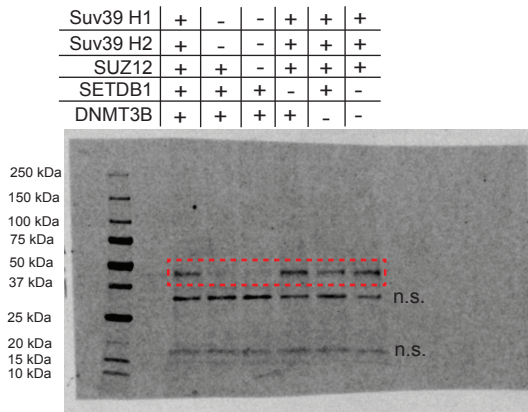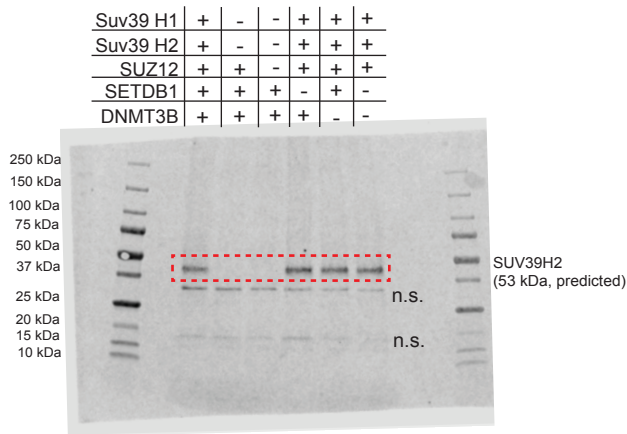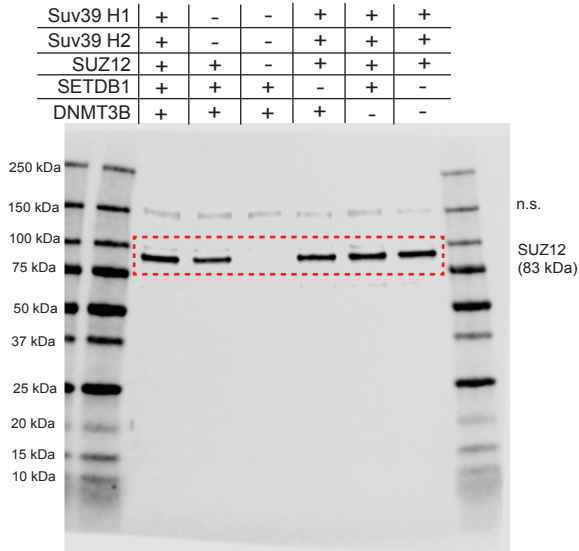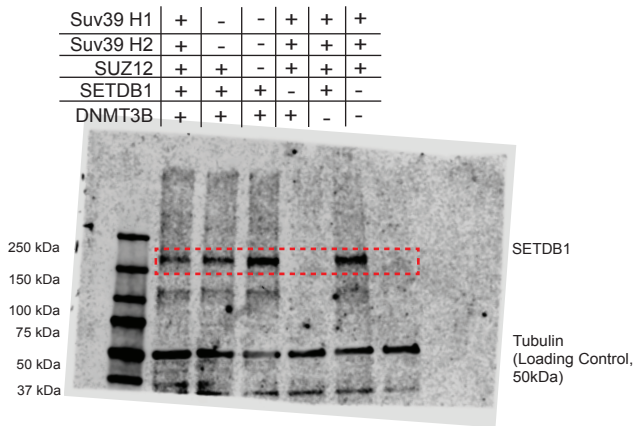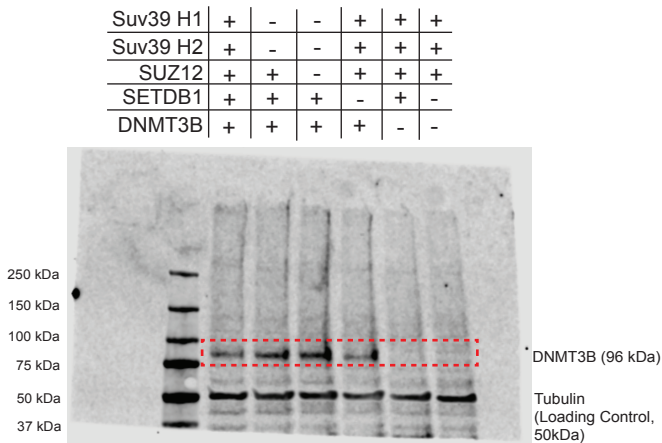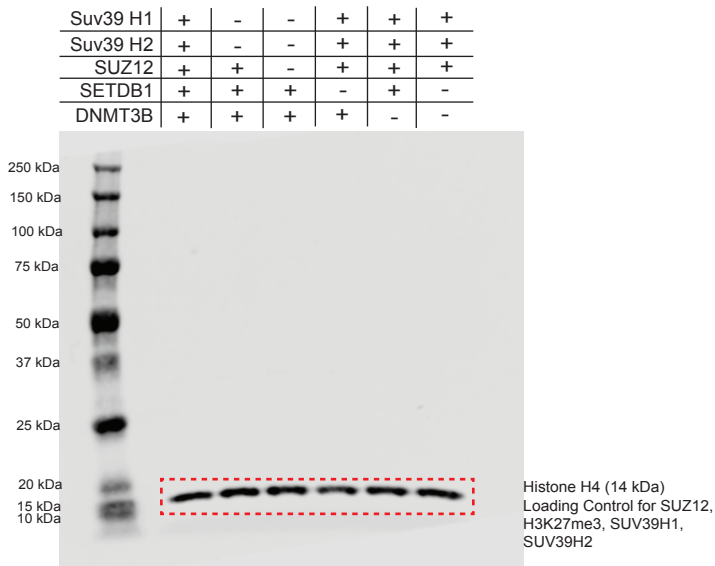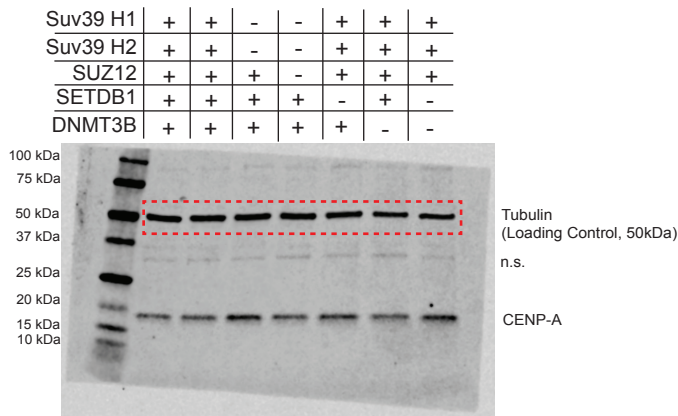

Supplement: Supplementary file 8 — Uncropped blots. [file 41594_2025_1706_MOESM8_ESM.pdf]
