# Supplementary material for: Heterochromatin boundaries maintain centromere position, size and number
Source: Nat Struct Mol Biol. Author manuscript; Available in PMC 2025 Dec 5. (PMC7618434; doi:10.1038/s41594-025-01706-2)
Supplement: Supplementary file 2 [file EMS211245-supplement-supplementary_tables.pdf]

nature structural & molecular biology

Supplementary information https://doi.org/10.1038/s41594-025-01706-2

# Heterochromatin boundaries maintain centromere position, size and number

In the format provided by the authors and unedited

## Supplemental Tables

| Cell Line | Chromosome | Start | End |
|---|---|---|---|
| Parent 100d Clone 1.2 | Chr21 (Hap1) | 4830300 | 4895300 |
| *SUV39H1Δ/H2Δ/SUZ12Δ* | Chr1 (Hap1) | 125004534 | 125149534 |
| *SUV39H1Δ/H2Δ/SUZ12Δ* | Chr1 (Hap1) | 126269534 | 126354534 |
| *SUV39H1Δ/H2Δ/SUZ12Δ* | Chr9 (Hap2) | 46215681 | 46365681 |
| *SUV39H1Δ/H2Δ/SUZ12Δ* | Chr20 (Hap1) | 27068226 | 27113226 |
| *SUV39H1Δ/H2Δ/SUZ12Δ* | Chr21 (Hap1) | 4650300 | 4860300 |
| *SUV39H1Δ/H2Δ/SUZ12Δ* 100d Clone 1.2 | Chr1 (Hap1) | 125109534 | 125149534 |
| *SUV39H1Δ/H2Δ/SUZ12Δ* 100d Clone 1.2 | Chr1 (Hap1) | 126269534 | 126419534 |
| *SUV39H1Δ/H2Δ/SUZ12Δ* 100d Clone 1.2 | Chr3 (Hap2) | 96531917 | 96561917 |
| *SUV39H1Δ/H2Δ/SUZ12Δ* 100d Clone 1.2 | Chr9 (Hap2) | 46215681 | 46360681 |
| *SUV39H1Δ/H2Δ/SUZ12Δ* 100d Clone 1.2 | Chr20 (Hap1) | 27063226 | 27128226 |
| *SUV39H1Δ/H2Δ/SUZ12Δ* 100d Clone 1.2 | Chr21 (Hap1) | 4645300 | 4865300 |
| *SETDB1Δ* | Chr7 (Hap1) | 65533216 | 65578216 |
| *SETDB1Δ* | Chr9 (Hap1) | 45447340 | 45487340 |
| *SETDB1Δ* | Chr19 (Hap2) | 29111633 | 29296633 |
| *SETDB1Δ* | Chr20 (Hap2) | 27113480 | 27193480 |
| *SETDB1Δ* | Chr21 (Hap1) | 4825300 | 4895300 |
| *DNMT3BΔ* | Chr9 (Hap2) | 46100681 | 46170681 |
| *DNMT3BΔ* | Chr21 (Hap1) | 4830300 | 4895300 |
| *SETDB1Δ/ DNMT3BΔ* | Chr9 (Hap2) | 49140681 | 49325681 |
| *SETDB1Δ/ DNMT3BΔ* | Chr19 (Hap2) | 29116633 | 29321633 |
| *SETDB1Δ/ DNMT3BΔ* | Chr20 (Hap2) | 27088480 | 27188480 |
| *SETDB1Δ/ DNMT3BΔ* | Chr21 (Hap1) | 4835300 | 4900300 |

**Table 1:** Summary of the coordinates of all new CENP-A containing CDRs within HOR regions as defined by CENP-A DiMeLo-seq.

| Vector | Target | Forward | Reverse |
|---|---|---|---|
| pLentiCRISPRv2-Blast | *SUV39H1* | CACCGGATCTTCTTGTAATCGCAC | AAACGTGCGATTAACAAGAAGATCC |
| pLentiCRISPRv2-Blast | *SUV39H2* | CACCGTTTCGAACTAGCAATGGACG | AAACCGTCCATTGCTAGTTCGAAAC |
| pLentiCRISPRv2-Blast | *SETDB1* | CACCGCCTTCCTGGGTGCATTGGTT | AAACAACCAATGCACCCAGGAAGGC |
| pLentiCRISPRv2-Blast | *DNMT3B* | CACCGAGAGTCGCGAGCTTGATCTT | AAACAAGATCAAGCTCGCGACTCTC |
| PX458 | *SUZ12* gRNA 1<br>*SUZ12* gRNA 2 | CACCGCAGTTCACTCTTCGTTGGAC<br>CACCGCAGTTCACTCTTCGTTGGAC | AAACAGGAGCCGTAGATTTATCATC<br>AAACGTCCAACGAAGAGTGAACTGC |

**Table 2:** Table of gRNA Primers used for CRISPR knockouts. Bsmbi and Bbsi compatible overhangs are underlined.

| Target | Antibody | Working dilution | Assay |
|---|---|---|---|
| SUV39H1 | Cell Signalling (D11B6) | 1:1000 | Immunoblot |
| SUV39H2 | Abcam (ab190870) | 1:1000 | Immunoblot |
| SUZ12 | Cell Signalling (D39F6) | 1:1000 | Immunoblot |
| SETDB1 | Cell Signalling (C1C12) | 1:1000 | Immunoblot |
| CENP-A | Cell Signalling (2186S) | 1:1000 | Immunoblot |
| Histone H4 | Merck Millipore (05-858) | 1:1000 | Immunoblot |
| DNMT3B | Cell Signalling (E2Q3Z) | 1:800 | Immunoblot |
| α-tubulin | Sigma (T9026) | 1:5000 | Immunoblot |
| H3K9me3 | Abcam (ab8898) | 1:1000 | Immunoblot |
| H3K27me3 | Cell Signalling (C36B11) | 1:1000 | Immunoblot |
| Rabbit IgG | IRDye® 800CW (926-32211) | 1:5000 | Immunoblot |
| Mouse IgG | Rockland DyLight™ 680 (610-744-124) | 1:5000 | Immunoblot |
| Rabbit IgG | Jackson ImmunoResearch HRP (111-035-008) | 1:5000 | Immunoblot |
| CENP-A | Enzo (ADI-KAM-CC006) | 1:50 @ 1mg/mL | DiMeLo-Seq |
| H3K9me3 | Abcam (ab8898) | 1:50 @ 1mg/mL | DiMeLo-Seq |
| H3K27me3 | Cell Signalling (C36B11) | 1:50 @ 0.1mg/mL | DiMeLo-Seq |
| CENP-C | Gift from Don Cleveland, described in Mitra *et al*, 2020 *Nat Comms*; (Human CENP-C aa1-198, Mouse IgG1k) | 1:50 @ 1mg/mL | DiMeLo-Seq |
| Mouse IgG1 | Cell Signalling (G3A1) | 1:125 @ 2.5mg/mL | DiMeLo-Seq |
| Rabbit IgG | Cell Signalling (2729) | 1:500 @ 1mg/mL | DiMeLo-Seq |
| CENP-A | Enzo (ADI-KAM-CC006) | 1:50 @ 1mg/mL | CUT&RUN |
| H3K9me3 | Abcam (ab8898) | 1:50 @ 1mg/mL | CUT&RUN |
| H3K27me3 | Cell Signalling (C36B11) | 1:50 @ 0.1mg/mL | CUT&RUN |
| Rabbit IgG | EpiCypher (13-0042) | 1:50 @ 0.5,g/mL | CUT&RUN |

**Table 3:** Antibodies used for Immunoblotting, DiMeLo-Seq and CUT&RUN.
